# Enrichment of Phosphorylated Tau (Thr181) and Functionally Interacting Molecules in Chronic Traumatic Encephalopathy Brain-derived Extracellular Vesicles

**DOI:** 10.14336/AD.2020.1007

**Published:** 2021-09-01

**Authors:** Satoshi Muraoka, Weiwei Lin, Kayo Takamatsu-Yukawa, Jianqiao Hu, Seiko Ikezu, Michael A DeTure, Dennis W Dickson, Andrew Emili, Tsuneya Ikezu

**Affiliations:** ^1^Department of Pharmacology & Experimental Therapeutics, Boston University School of Medicine, Boston, MA, USA.; ^2^Department of Biochemistry, Boston University, Boston, MA, USA.; ^3^Center for Network Systems Biology, Boston University, Boston, MA, USA.; ^4^Department of Neuroscience, Mayo Clinic, Jacksonville, FA, USA.; ^5^Center for Systems Neuroscience, Boston University, Boston, MA, USA.; ^6^Department of Neurology and Alzheimer’s Disease Center, Boston University School of Medicine, Boston, MA, USA.

**Keywords:** chronic traumatic encephalopathy, extracellular vesicles, microtubule-associated protein tau, plexin-A4, proteome, synaptosomal-associated protein 25, tauopathy, ubiquitin-like modifier-activating enzyme 1

## Abstract

Chronic Traumatic Encephalopathy (CTE) is a tauopathy that affects individuals with a history of mild repetitive brain injury. The initial neuropathologic changes of CTE include perivascular deposition of phosphorylated microtubule-associated protein tau (p-tau). Extracellular vesicles (EVs) are known to carry pathogenic molecules, such as tau in Alzheimer’s disease and CTE suggesting their contribution in pathogenesis. We therefore examined the protein composition of EVs separated from CTE and an age-matched control brain tissues by tandem mass tag -mass spectrometry. The reporter ion intensity was used to quantify the identified molecules. A total of 516 common proteins were identified among three sets of experiments. Weighted protein co-expression network analysis identified 18 unique modules of co-expressed proteins. Two modules were significantly correlated with total tau (t-tau) and p-tau protein in the isolated EVs and enriched in cellular components and biological processes for synaptic vesicle secretion and multivesicular body-plasma membrane fusion. The p-tau (Thr181) level is significantly higher in CTE EVs compared to control EVs and can distinguish the two groups with 73.6% accuracy. A combination of t-tau or p-tau (Thr181) with SNAP-25, PLXNA4 or UBA1, enhanced the accuracy to 96.3, 93.8 and 93.8%, respectively. Bioinformatic protein-protein interaction analysis revealed the functional interaction of SNAP-25 and PLXNA4 with tau, suggesting their interaction in CTE EVs. These data indicate the future application of identified EV proteins for monitoring the CTE risk assessments and understanding the EV-mediated disease progression mechanism.

Orginal Article

Chronic Traumatic Encephalopathy (CTE) is a progressive neurodegenerative disease associated with repetitive head impacts such as occur with boxers, hockey players, and American football players [1, 2]. CTE is considered one of the tauopathies marked by an accumulation and deposition of phosphorylated microtubule-associated tau (p-tau) protein [3]. The initial neuropathologic changes of CTE include perivascular deposition of p-tau as neurofibrillary tangles and neuritic inclusions at the sulcal depths of the superior, dorsolateral, and inferior frontal cortices [4-6]. At this time, CTE can only be diagnosed by neuropathologic examination. However, a recent study reported preliminary support for the use of the positron emission tomography (PET) p-tau ligand flortaucipir to detect CTE in living former national football league (NFL) players [7]. It has been recently reported that the conformation of tau filaments in CTE is distinct from Alzheimer’s disease (AD) [8]. Although the onset of disease is mostly attributed to the concussion or head injury, the exact mechanism of disease spread, especially the propagation of misfolded tau, is yet to be fully understood [9,10].

Extracellular vesicles (EVs), including exosomes (50 - 150 nm), microvesicles (150 - 1000 nm), and apoptotic bodies (1000 - 5000 nm) are secreted from almost any cell types, including neuron, glia, endothelial cells, into the extracellular space [11,12]. EVs contain protein, lipid, glycosaminoglycans and nucleic acids (including mRNAs and ncRNAs) from the original cells, which could be transferred for cell-to-cell communication. In the context of neurodegenerative disorders, amyloid-β peptide (Aβ), α-synuclein, prion protein, superoxide dismutase 1, TAR DNA-binding protein-43 and tau are reported in EVs separated from blood, cerebrospinal fluid (CSF) and brain tissues [13-16]. EVs released from microglia play a significant role in spreading tau and α-synuclein [17,18], and intracerebroventricular injection of tau or α-synuclein-containing EVs can initiate pathological development in mouse brains [18, 19]. We have previously shown that tau level is increased in the plasma EV of former football players with risk of CTE [20]. Our more recent study detected t-tau and p-tau in the CSF EV of the same cohort, although there was no difference compared to the healthy controls [21], suggesting that characterization of EVs directly isolated from CTE brain may shed light on the molecular composition and their interaction with tau to understand their potential role in CTE development. Here, we for the first time examined the proteomic profiles of EVs separated from the brain tissue of CTE cases and an age-matched control (CTRL) group with no history of contact sports.

## MATERIALS AND METHODS

### Human tissue samples

The unfixed frontal cortical brain tissue samples were obtained from Mayo Clinic (four CTE and four control samples) and through NIH NeuroBioBank, including University of Miami (one CTE and two control samples), McLean Hospital (two CTE and four control samples), University of Maryland (one CTE). The samples were matched for age and sex (Table 1). The study was approved by the Institutional Review Board at Boston University School of Medicine, Mayo Clinic and NeuroBioBank-participating institutions with informed consent. Group comparisons of age and the post-mortem interval (PMI) were performed using independent *t*-tests.

**Table 1:** Patient information

	Control (n=10)	CTE (n=8)	*t*-test^a^	*p*-value^b^
**Gender**	10 Males	8 Males		
**Age, mean**	65.3 ± 6.67	61.4 ± 14.47	-0.748	0.466
**Post-mortem interval (PMI), mean**	20.79 ± 8.19	21.8 ± 3.39	0.163	0.876

a The group comparisons were performed using independent t-test.

b The statistical significance of the differences were calculated using a two-tailed test.

### Purification of EVs from human brain samples

Grey matter tissues from the frontal cortex of deceased CTE and control cases (0.5 g per sample) were processed for EV extraction based on our reported method with modifications [22,23]. Briefly, frozen brain tissue was chopped on ice using a razor blade (#12-640 Fischer Scientific) to generate approximately 0.5 mm-wide pieces. The sections were transferred to 3mL of Hibernate E solution (# A1247601 Gibco) containing 20 U of papain (#LK003178 Worthington-biochemical corporation) in Earle’s Balanced Salt Solution (EBSS) (#14155063 Gibco) and then incubated at 37°C for 15 min by stirring once every 5 min. After the incubation, the samples were placed on ice, and added with 6 mL of ice-cold Hibernate E solution supplemented with Halt™ Protease and Phosphatase Inhibitor Cocktails (#PI78443 Fisher scientific). The samples were gently homogenized (20 strokes) with a glass-Teflon homogenizer (#89026-384 VWR), and filtered with 40-μm mesh filter (# 22-363-547 Fisher scientific), followed by centrifugation at 300 × *g* for 10 min at 4°C (#5720R Eppendorf). The supernatant was transferred to a new 15-mL polypropylene tube and centrifuged at 2,000 × *g* for 10 min at 4°C (# 5720R Eppendorf). The supernatant was transferred to a 30-mL conical tube and centrifuged at 10,000 × *g* for 10 min at 4°C (#Avanti J-E JA25-50 Beckman Coulter). The supernatant filtered through a 0.22-μm polyethersulfone membrane filter (# SLGP033RS EMD Millipore) into new a polyallomer ultracentrifuge tube with 13.2-mL capacity (#331372 Beckman Coulter), diluted with double-filtered phosphate-buffered saline (dfPBS) with 0.22-μm polyethersulfone membrane filter to 12 mL, and centrifuged at 100,000 × *g* for 70 min at 4°C (#Optima-XE SW41 Beckman Coulter). The pellet was resuspended in 2 mL of 0.475M of sucrose solution (# S5-3 Fisher science) in dfPBS. The sucrose step gradient was created in dfPBS with six 2-mL steps starting from 2.0M to 1.5M, 1.0M, 0.825M, 0.65M, and 0.475M (containing the resuspended pellet) in a polyallomer ultracentrifuge tube. Each layer was colored with commercially available food coloring to facilitate capture of the EV-rich interphase present between certain steps. The gradient was centrifuged at 200,000 × *g* for 20 h at 4°C (35,000 rpm with # Optima-XE SW41 Beckman Coulter). The gradient was collected in 2-mL fractions, except for the first and last fractions, which were 1 mL each. The interphases between the second (0.65M) and third (0.825M) steps correspond to fraction “V” and the third and fourth steps corresponded to fraction “VI” have a buoyant density of 1.10 - 1.12 and 1.12 - 1.15 g/cm^3^, respectively, and enriched in EVs. The V and VI fractions were mixed, diluted to a total volume of 12 mL with dfPBS and centrifuged at 100,000 × *g* for 70 min at 4°C (# Optima-XE SW41 Beckman Coulter). The final pellet was resuspended in 30μL of dfPBS as an EV-enriched sample.

### Protein concentrations

The bicinchoninic acid (BCA) assay was used to determine protein concentration for each sample using BCA protein assay kit (# 23225 Pierce) as previously described [23]. EVs were diluted 1:10 before loading into the assay, and a 1:8 ratio of sample to reaction components was used. All assays were allowed to incubate at 60°C for 30 min before protein concentration was read in at 562 nm (SynergyMix, Biotek).

### Enzyme-Linked Immunosorbent Assay (ELISA)

EVs were solubilized in guanidine hydrochloride buffer (8M Guanidine hydrochloride, 50mM Tris-HCl) supplemented with Halt™ Protease Inhibitor Cocktails and incubated at room temperature for 3 h with gentle agitation. After dilution of samples to 1:100 in dilution buffer, ELISAs were performed to assess levels of t-tau and p-tau (t-tau: # KHB0042 and pT181: # KHO0631, Thermo Fisher) according to manufacturer’s instructions.

### Nanoparticle Tracking Analysis (NTA)

All samples were diluted in dfPBS at least 1:1000 or more to get particles within the target reading range for the Nanosight 300 machine (Malvern Panalytical Inc), which is 10-100 particles per frame. Using a syringe pump infusion system (Harvard Laboratories/Malvern), five 60-s videos were taken for each sample at 21°C. Analysis of particle counts was carried out in the Nanosight NTA 3.2 software (Malvern Panalytical Inc) with a detection threshold of 5. Particle counts were normalized for dilution on the machine, dilution of the final pellet, and starting material for EVs extraction.

### Transmission electron microscopy (TEM)

The EV separated from CTE and control brain tissue were analyzed by TEM. The EV sample (5µl) was adsorbed for 1 min to a carbon-coated mesh grid (# CF400-CU, Electron Microscopy Sciences) that had been made hydrophilic by a 20-s exposure to a glow discharge (25 mA). Excess liquid was removed with a filter paper (#1 Whatman). The grid was then floated briefly on a drop of water (to wash away phosphate or salt), blotted on a filter paper, and then stained with 0.75% uranyl formate (# 22451 Electron Microscopy Sciences) for 30 s. After removing the excess uranyl formate, the grids were examined, and random fields were photographed using a JEOL 1200EX TEM with an AMT 2k CCD camera.

### Mass spectrometry

#### Sample preparation

The EV samples were disrupted with 8M Urea (Fisher Scientific). 5mM dithiothreitol (# 167956A, Fisher Scientific) was added to the samples, and total protein was reduced for 1 h at 37°C before alkylation with 5mM iodoacetamide (# A0381880 ACROS) for 30 min at room temperature in the dark. The samples were digested with sequencing grade trypsin (1:20 w/w trypsin-to-protein) after dilution to 1M urea with 50mM ammonium bicarbonate (pH 7.5) for 16 h at 37°C. The digested peptides were desalted using C18 ziptips (Fisher Scientific) and dried in a vacuum centrifugation (Fisher Scientific).

#### TMT labeling

The digested peptides were chemically labeled with isobaric tandem mass tag (TMT) stable-isotopes for quantitative proteomics analysis as previously described [23]. Briefly, identical amounts of peptides were resuspended in 0.1 M triethylammonium bicarbonate (TEAB) and incubated with the TMT 10-plex reagents (Thermo Scientific, 4/1, w/w) for 1 h at room temperature. 8 μL of 5% hydroxylamine was added to quench the reactions. Equal amounts of labeled samples were combined and desalted using C18 tips before suspension in 0.1% formic acid-2% acetonitrile-water prior to analysis.

**Figure 1.**
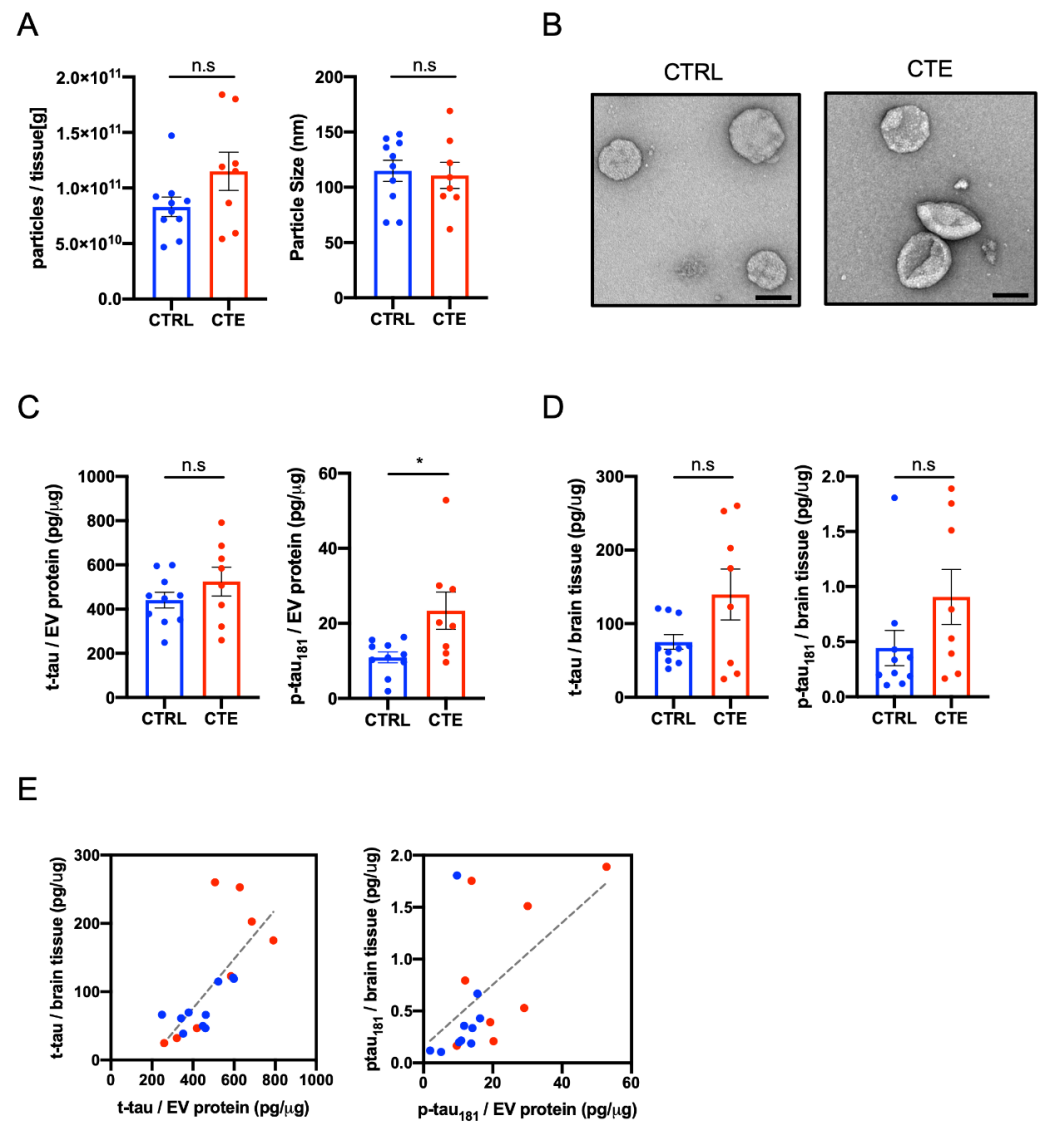
**Biochemical characteristic of brain-derived EVs separated from CTE brain tissue**. (**A**) Left: Particle numbers of brain-derived EV fraction from CTRL and CTE by Nanoparticle tracking analysis (*p* = 0.1522 by Mann-Whitney test). Right: Particle size of brain-derived EV fraction (*p* = 0.6480). (**B**) Transmission electron microscopy (TEM) image of CTRL and CTE brain-derived EV fraction. Left: CTRL, Right: CTE. Scale bar; 100nm. (**C**) T-tau and tau phosphorylated at threonine 181 (p-tau_181_) levels in CTRL and CTE brain-derived EVs by ELISA. Left: brain-derived EV t-tau (*p* = 0.3599). Right: brain-derived EV p-tau_181_ (*p* = 0.0266). (**D**) T-tau and p-tau_181_levels in CTRL and CTE brain tissue homogenates by ELISA. Left: brain tissue homogenates t-tau (*p* = 0.3154). Right: brain tissue homogenates p-tau_181_ (*p* = 0.1220). (**E**) Scattered plot of brain-derived EV and brain tissue homogenates. Left: t-tau (r = 0.7283, *p* = 0.0006 using two-tailed t-test), Right: (r = 0.5449, *p* = 0.0194).

#### Liquid chromatography (LC)-electrospray ionization (ESI) tandem mass-spectroscopy (MS/MS) Analysis

LC-ESI-MS/MS analysis was performed on an Easy nanoLC1200 (Thermo Fisher Scientific) coupled to a Q-Exactive HF-X mass spectrometer (Thermo Fisher Scientific). 2 µg of the pooled, labeled peptides were loaded onto a C18 pre-column connected to an easy spray C18 analytical column (2 mm, 75 mm × 50 cm, Thermo Fisher Scientific) and analyzed using positive mode by data dependent acquisition. The full scan range was set to 300 to 1650 m/z with an AGC of 3e^6^ followed by 15 MS/MS scans. The normalized collision energy was set to 32% with a dynamic exclusion window of 40 s. The mobile phase was 2% ACN with 0.1% FA (A) and 80% ACN with 0.1% FA (B). The gradient profile was 5% B at 0 min, 30% B at 90 min, 100% B from 99 to 116 min, and 2% B at 117 to 120 min. The column oven was set to 55°C.

#### Sequence database

The raw MS/MS spectra data were searched using MaxQuant (v1.6.7) (Max Planck Institute of Biochemistry) against the Uniprot/Swissprot database for Homo sapiens with a 1% estimated protein-level false discovery rate (FDR). For protein identification, two missed trypsin cleavage sites were allowed. Carbamidomethylation of cysteine residues was set as a fixed modification, while methionine oxidation and N-terminal acetylation were set as variable modifications.

### Weighted protein correlation network analysis (WPCNA)

WPCNA was performed based on the original weighted gene correlation network analysis as described [24].

### Statistical Analysis

Statistical analysis was conducted using IBM SPSS software ver.25 and GraphPad Prism8 (GraphPad Software, CA, USA). Data were analyzed by t-test or, when appropriate, a nonparametric Mann-Whitney t-test. Group comparisons of age and the post-mortem interval (PMI) were performed using independent *t*-tests. The Gene Ontology of identified proteins were elucidated by the Database for Annotation, Visualization and Integrated Discovery (DAVID) Bioinformatics Resources 6.8 [25,26]. The Protein networks and pathway analysis were generated using Ingenuity Pathway Analysis (IPA) (version 01-12) (https://www.qiagenbioinformatics.com/products/ingenuity-pathway-analysis/). The Venn diagram and heatmap analysis were generated using Venny_2.1 (http://bioinfogp.cnb.csic.es/tools/venny/) and ClustVis (https://biit.cs.ut.ee/clustvis/).

**Table 2:** Up- and down-regulated brain-derived EV proteins in CTE compared with controls

Uniprot ID	Gene Name	Control Average a	CTE Average	log_2_(CTE/Controls)	*p* value b
**P05408**	SCG5	1.062	0.786	-0.435	0.0268
**P27338**	MAOB	1.061	0.904	-0.231	0.0498
**P30101**	PDIA3	1.023	0.916	-0.160	0.0161
**P31946**	YWHAB	0.579	0.449	-0.368	0.0291
**P10599**	TXN	0.983	1.083	0.140	0.0302
**P31150**	GDI1	0.969	1.034	0.094	0.0279
**Q13303**	KCNAB2	0.931	1.095	0.234	0.0408
**P50991**	CCT4	1.135	1.389	0.291	0.0418
**Q99747**	NAPG	1.008	1.168	0.213	0.0392
**P19784**	CSNK2A2	0.918	1.027	0.161	0.0178
**Q9HCH3**	CPNE5	0.939	1.050	0.161	0.0313
**P45974**	USP5	0.916	1.071	0.225	0.0353
**Q4G0F5**	VPS26B	0.985	1.117	0.183	0.0453
**Q7Z6L0**	PRRT2	0.905	1.155	0.353	0.0184
**P60880**	SNAP-25	0.981	1.065	0.119	0.0365
**Q14194**	CRMP1	1.056	1.236	0.227	0.0095
**Q2M1P5**	KIF7	1.016	1.145	0.173	0.0161
**P05023**	ATP1A1	1.021	1.096	0.103	0.0409
**P09471**	GNAO1	0.949	1.093	0.204	0.0389
**P28482**	MAPK1	0.954	1.148	0.266	0.0204
**Q99962**	SH3GL2	0.981	1.116	0.186	0.0436
**Q01813**	PFKP	1.086	1.235	0.186	0.0080
**P22314**	UBA1	0.959	1.053	0.134	0.0167

a The value shows normalized intensity by standard pooled sample.

b The statistical significance of the differences were calculated using Student’s test.

## RESULTS

### Biochemical and biological of EVs isolated from CTE and control brain tissues

The EVs were separated from eight CTE brain tissues and an age-matched ten control group using the discontinuous sucrose gradient ultracentrifugation as previously reported [22,23]. The purities of EVs separated from brain were checked by LC-EMS-MS/MS [11,22,23]. We found that the EV fraction was presented in tetraspanin, annexins, endosomal sorting complexes required for transport (ESCRT) complexes including vacuolar sorting (VPS) protein and ras-related protein Rab family, and non-EV molecules such as 94 kDa glucose-regulated protein (Grp94) and endoplasmic reticulum chaperone BiP as listed in MISEV2018 guidelines [11] (Supplementary Table 1). The concentrations of particles were not significantly different in separated EV fraction between CTE and CTRL (*p* = 0.1522) (Fig. 1A). The size distributions of EVs were not significantly different between these groups (*p* = 0.6480) (Fig. 1A). Figure 1B represents separated EVs was shown by TEM. We measured the levels of t-tau and p-tau at threonine 181 (p-tau_181_) in lysed EVs by ELISA. There was no difference in the EV t-tau levels between the CTE group and the control group (*p* = 0.3599) (Fig. 1C). The p-tau_181_ in EVs was significantly increased in CTE compared to control group (*p* = 0.0266) (Fig. 1C). The amounts of t-tau and p-tau in brain tissue homogenate were quantified by ELISA, and we analyzed the correlation between the homogenate samples and EV samples. There was no significant difference in t-tau and p-tau_181_ in the brain tissue homogenates between CTE and CTRL (t-tau; *p* = 0.3154, p-tau_181_; *p* = 0.1220) (Fig. 1D). In addition, there was a significantly positive correlation between the brain tissue homogenates and their EVs (t-tau; r = 0.7283, *p* = 0.0006, p-tau_181_; r = 0.5449, *p* = 0.0194) (Fig. 1E). These data demonstrate that p-tau_181_ is significantly enriched in CTE EVs compared to CTE brain homogenate and suggests the diagnostic potential of p-tau_181_ in CTE EVs.

**Figure 2.**
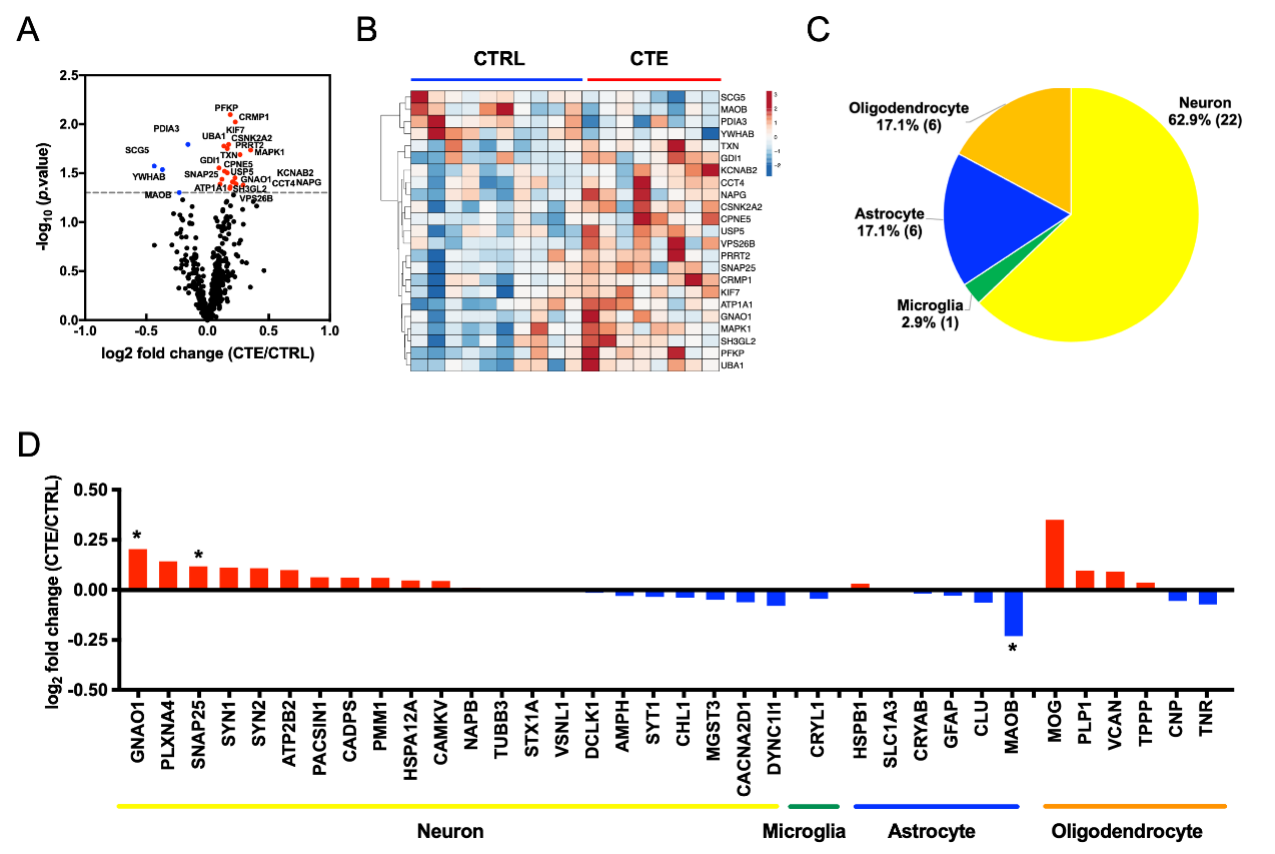
**Proteome profiling of CTE and control brain-derived EV**. (**A**) Volcano plot showing degree of differential expression of brain-derived EV proteins in CTE compared with CTRLs. The x-axis indicates log_2_ transformed fold change in expression. The y-axis shows -log_10_ transformed *p*-values. The grey dot line shows the 1.3010 -log_10_(*p*-value) cutoff. (**B**) Heatmap of 23 proteins with the 1.3010 -log_10_(*p*-value) cutoff. The value shows log_2_(fold change). (**C-D**) Cell type-specific proteins comparison of CTE and CTRL brain-derived EV. (**C**) Enrichment of brain cell type-specific markers in brain-derived EV proteins. Yellow: Neuron, Green: Microglia, Blue: Astrocytes, Orange: Oligodendrocytes. The parentheses show the number of identified cell type-specific proteins. (**D**) Comparison of the cell type-specific protein in CTE-derived EVs and CTRL EVs. The red bar shows higher expression in CTE compared with CTRL and Blue bar indicates higher expression in CTRL compared with CTE. The asterisk shows molecules with significantly difference. *p < 0.05 as determined by Student’t *t*-test.

**Table 3:** Canonical pathway for the 23 differentially expressed EV proteins by Ingenuity pathway analysis.

Canonical pathways	*p*-value	Overlap
**Melatonin Signaling**	5.15E-05	4.2 % (3/72)
**Insulin Secretion Signaling Pathway**	9.61E-05	1.6 % (4/243)
**IGF-1 Signaling**	1.54E-04	2.9 % (3/104)
**Antioxidant Action of Vitamin C**	1.77E-04	2.8 % (3/109)
**14-3-3-mediated Signaling**	2.78E-04	2.4 % (3/127)

### Proteome profiling of CTE and control brain-derived EVs

TMT labeling-based quantitative analysis was performed by MaxQuant software. A total of 516 proteins were identified and quantified in common among all three TMT-based analyses of CTE and control brain-derived EVs (Supplementary Table 2). The functionality of the identified proteins was analyzed by Gene Ontology analysis using the Database for Annotation, Visualization and Integrated Discovery (DAVID) software tool (Supplementary Fig. 1) and by Ingenuity Pathway Analysis (IPA) (Supplementary Fig. 2). We observed significant enrichment of proteins previously linked to extracellular exosome molecules (cellular component), protein-binding molecules (molecular function), cell-cell adhesion (biological process), brain (tissue expression ontology), schizophrenia (disease ontology) and synaptic vesicle cycle (KEGG pathway) by DAVID GO in the common molecules (Supplementary Fig. 1). We also detected significant enrichment of components involved in synaptogenesis signaling and Huntington’s disease by IPA (Supplementary Fig. 2). Volcano plot analysis showed 19 proteins were significantly upregulated, while 4 proteins were significantly down-regulated in CTE compared to the control groups (Fig. 2A, Table 2 and Supplementary Fig. 3). The expression levels of these 23 proteins in each patient samples are displayed in a heatmap (Fig. 2B). The enriched pathway analysis for 23 differentially expressed EV proteins by Ingenuity pathway analysis were enriched the Insulin Secretion Signaling pathway, IGF-1 Signaling and 14-3-3-mediated signaling, which reported to relate with neurodegenerative diseases, including AD and CTE [27-30] (Table 3). In addition, Upstream effect analysis predicted APP (*p* = 1.46E-08), MAPT (Tau, *p* = 2.02E-06), PSEN1 (Presenilin-1, *p* = 2.45E-06) and MKNK1 (MAPK Interacting Serine/Threonine Kinase 1, *p* = 3.37E-06) as an upstream regulator of the 23 proteins, suggesting the AD/CTE-related pathology for the enhanced levels of these molecules in CTE brain EVs.

We assessed brain cell-type-specific molecules in the EV proteomics dataset using the mouse brain proteome database [31]. The distribution of brain cell-type specific markers indicates that in the human brain, 62.9% of identified EV molecules are likely of neuronal origin, while 37.1% are of glia origin including microglia, astrocytes and oligodendrocytes (Fig. 2C). The neuron cell type-specific molecules were enriched in CTE EVs, while glia cell type molecules were unchanged (Fig. 2D). This is different from AD brain-derived EVs, which are enriched in glia-specific molecules while neuron-specific molecules are down-regulated [22], suggesting the difference in the origin of EVs between CTE and AD brains.

### Protein co-expression network and differential abundance of t-tau and p-tau in CTE brain EVs

**Figure 3.**
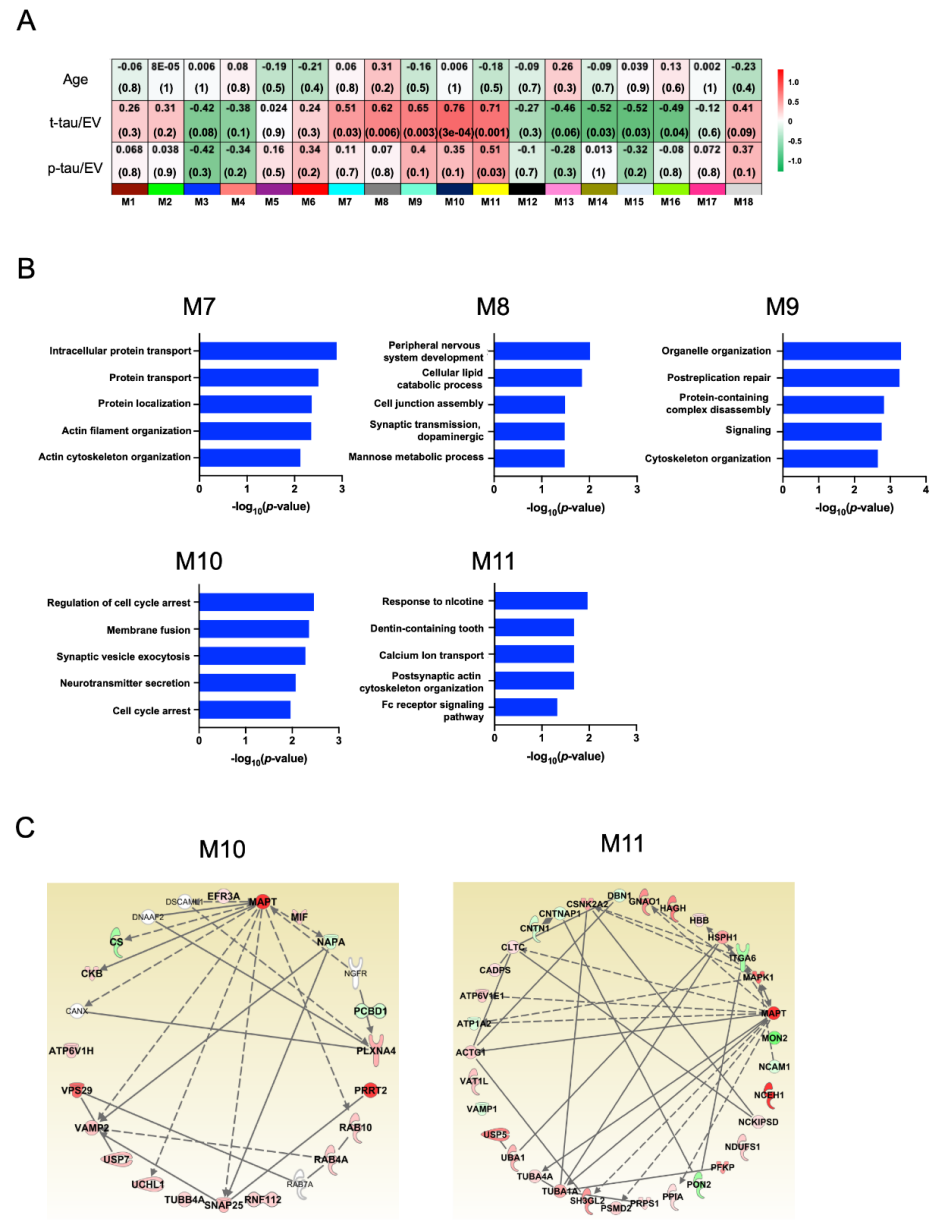
**Protein co-expression Network and differential abundance by t-tau and p-tau in CTE brain EVs**. Weighted Protein Correlational Network Analysis (WPCNA) was analyzed on eight CTE and ten control. (**A**) The Heatmap show the correlation of t-tau and p-tau. The red color indicates positive correlation, and the green shows negative correlation. (**B**) Two module which indicated significant correlation with t-tau and p-tau were analyzed Cellular component, Molecular Function, and Biological Process by GO analysis. (**C**) The protein Network analysis was performed two module and tau by IPA-based. The intensity of the node color shows the degree of up-regulation (red) or down- regulation (green).

We performed weighted protein co-expression network analysis (WPCNA) on brain-derived EVs to identify co-expression molecules which were correlated with t-tau and p-tau in EVs. The WPCNA identified 18 putative modules. Among these, five modules and one module were significantly positively and negatively correlated with t-tau and p-tau in EVs, respectively (Fig. 3A, Table 4, 5, and supplementary Table 3).

To assess the biological process associated with these modules, we performed functional enrichment analysis for the positively correlated modules. These were characterized by the top GO term “Intracellular protein transport” for M7 module, “Peripheral nervous system development” for M8, “Organelle organization” for M9, “Cell cycle arrest” for M10 and “Response to nicotine” for M11 (Fig. 3B, Supplementary Table 3 and 4). The M10 module was most significantly correlated with t-tau (r = 0.76, *p* = 0.00003), and M11 module appeared with p-tau (r = 0.51, *p* = 0.03) (Fig. 3A). The 18 proteins included in the M10 module and 32 proteins included in M11 were assessed the protein-protein network analysis (Fig. 3C). We observed that subset of components in each module were previously reported to directly interact with tau protein.

**Figure 4.**
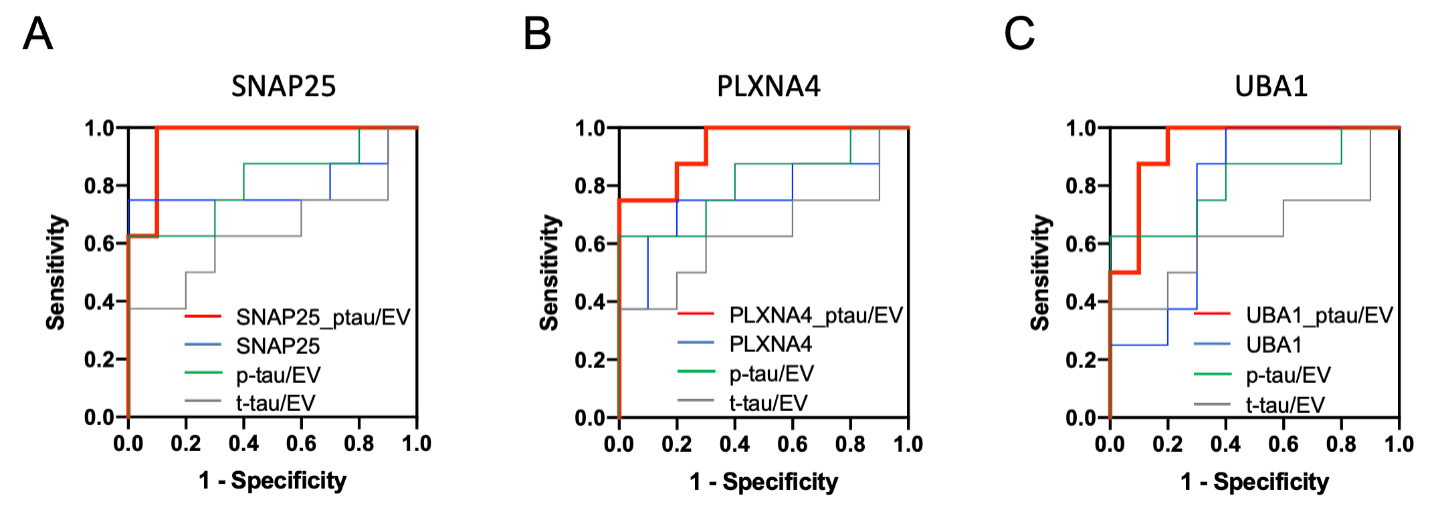
**Assessment of risk prediction for CTE with ROC curve: A ROC of possible pairs of top3 candidate proteins**. (**A**) SNAP-25; Area under ROC for single-marker (t-tau; Grey line, p-tau; Green line, SNAP-25; Blue line) were 0.638, 0.813 and 0.800, in multi-marker (p-tau and SNAP-25; Red line) was 0.963. (**B**) PLXNA4; for single-marker (t-tau; Grey line, p-tau; Green line, PLXNA4; Blue line) were 0.638, 0.813 and 0.763, in multi-marker (p-tau and SNAP-25; Red line) was 0.938. (**C**) UBA1; for single-marker (t-tau; Grey line, p-tau; Green line, PLXNA4; Blue line) was 0.638, 0.813 and 0.775, in multi-marker (p-tau and SNAP-25; Red line) was 0.938.

### Assessment of risk prediction for CTE with receiver operating characteristic curve

The 18 and 32 proteins included in M10 and M11 module were tested with t-tau or p-tau for their diagnostic potential by receiver operating characteristic (ROC) area under curve (AUC) analysis (Supplementary Table 5 and 6). We found that the true positive rate of the SNAP-25, PLXNA4, or UBA1 with p-tau were significantly higher than either one of molecules alone, resulting in an AUC of 0.963, 0.938 and 0.938, respectively (Fig. 4 and Table 6). The combination of each molecule with p-tau protein may serve as potential biomarkers for monitoring CTE development.

**Table 4:** Protein list of M10 module correlated with t-tau protein.

Uniprot ID	Gene Name	Correlation coefficient a	-log_10_(*p* value) b
**Q9UBQ0**	VPS29	0.862	0.00000416
**Q93009**	USP7	0.769	0.000190
**Q9ULX5**	RNF112	0.749	0.000343
**Q7Z6L0**	PRRT2	0.667	0.00248
**P54920**	NAPA	-0.649	0.00356
**P04350**	TUBB4A	0.637	0.00443
**P61026**	RAB10	0.637	0.00447
**Q9UI12**	ATP6V1H	0.607	0.00759
**P09936**	UCHL1	0.584	0.0109
**Q9HCM2**	PLXNA4	0.582	0.0113
**P14174**	MIF	0.564	0.0148
**P12277**	CKB	0.563	0.0150
**P20338**	RAB4A	0.551	0.0178
**O75390**	CS	-0.519	0.0271
**P60880**	SNAP-25	0.517	0.0279
**Q14156**	EFR3A	-0.503	0.0335
**P63027**	VAMP2	0.297	0.231
**P61457**	PCBD1	-0.281	0.258

a The correlation coefficient values were calculated by correlation analysis by R.

b The statistical significance of the differences were calculated using two-tailed test.

**Table 5:** Protein list of M11 module correlated with p-tau protein.

Uniprot ID	Gene Name	Correlation coefficient^a^	-log_10_ (*p* value)^b^
**Q6PIU2**	NCEH1	0.673	0.00222
**P09471**	GNAO1	0.665	0.00258
**Q01813**	PFKP	0.639	0.00430
**Q13200**	PSMD2	0.639	0.00433
**Q92598**	HSPH1	0.630	0.00504
**P28482**	MAPK1	0.548	0.0185
**P60891**	PRPS1	0.534	0.0223
**Q16775**	HAGH	0.511	0.0302
**Q15165**	PON2	-0.510	0.0307
**P22314**	UBA1	0.427	0.0772
**P36543**	ATP6V1E1	0.426	0.0779
**Q9HCJ6**	VAT1L	0.405	0.0956
**P23229**	ITGA6	-0.397	0.103
**Q99962**	SH3GL2	0.386	0.114
**P62937**	PPIA	0.381	0.118
**Q12860**	CNTN1	-0.381	0.119
**P63261**	ACTG1	0.373	0.1277
**Q9NZQ3**	NCKIPSD	0.349	0.155
**Q7Z3U7**	MON2	-0.344	0.162
**P68366**	TUBA4A	0.335	0.175
**P45974**	USP5	0.327	0.185
**P78357**	CNTNAP1	-0.315	0.204
**P28331**	NDUFS1	0.312	0.208
**P13591**	NCAM1	-0.290	0.244
**Q71U36**	TUBA1A	0.287	0.249
**P68871**	HBB	0.229	0.360
**P19784**	CSNK2A2	0.224	0.372
**Q00610**	CLTC	0.207	0.410
**Q9ULU8**	CADPS	0.116	0.647
**Q16643**	DBN1	0.107	0.674
**P23763**	VAMP1	0.067	0.790
**P50993**	ATP1A2	-0.040	0.876

a The correlation coefficient values were calculated by correlation analysis by R.

b The statistical significance of the differences were calculated using two-tailed test.

When we compare the current proteomics data to the AD brain-derived EV proteomics data [22], and found 613 proteins in common between CTE brain-derived EVs and AD brain-derived EVs (Supplementary Fig. 4A). The NDUFS1 and -8 showed a high positive correlation with CTE brain-derived EVs and with AD (Supplementary Fig. 4B). It is well known that mitochondrial dysfunction occurs in AD and may be an upstream inducer of tau phosphorylation [32,33]. In addition, mitochondrial dysfunction leads to their sorting to endolysosomal system and MVBs, which can release mitochondria-derived vesicles into the extracellular space as EVs [34]. Notably, 181 proteins were only upregulated in CTE group, which are enriched with Phagosome and Endocytosis Pathway as determined by DAVID GO. Of these, 290 were upregulated in both CTE and AD compared to control groups, while in pathway analysis revealed enrichment for Glutamatergic and GABAergic synapses annotations. These results suggest phagocytic activation of microglia and neuronal EV secretion CTE brains.

## DISCUSSION

In the present study, we identified a total of 516 proteins in CTE and control brain-derived EVs, which were enriched in p-tau_181_ and neuron-specific molecules in CTE group. The WPCNA revealed five modules that correlated with levels of t-tau and p-tau in EVs. Using the ROC and AUC, we have identified that combinations of p-tau_181_ with SNAP-25, PLXNA4 or UBA1 can distinguish CTE from control groups with 96.3, 93.8 and 93.8% accuracy, respectively. Protein-protein interaction analysis identified the functional interaction of tau with SNAP-25 and PLXNA4, suggesting their complex formation in CTE EVs.

The TMT-based quantitative proteomic analysis of brain-derived EV samples isolated from CTE patients found enriched neuron and oligodendrocyte-specific molecules in CTE brain-derived EVs, and identified PLXNA4, SNAP-25, and UBA1 as potential candidate molecules for monitoring the progression of CTE. Bi *et al.* recently reported a quantitative proteomics analysis of brain tissue samples from individuals neuropathologically diagnosed with CTE, and identified a total of 6218 proteins [35]. We found that 843 proteins are common between the proteome database of CTE brain-derived EVs and CTE brain tissue samples (Supplementary Fig. 5). In addition, we compared the quantitative value of the 23 differentially expressed EV proteins in the CTE brain tissue samples, and 3 molecules are significantly altered. CPNE5 showed a positive correlation between CTE brain EVs and tissue samples, while YWAHB and PFKP showed a negative correlation. Further study is necessary to understand the difference in the sorting mechanism of these molecules to EVs in the brain. They also indicated the neuron- and oligodendrocyte-derived molecules are downregulated in CTE [35]. The reduction of neuron- and oligodendrocyte-specific molecule in brain tissue may be reflected in the enrichment of these molecules in the brain EVs.

**Table 6:** Comparison of AUC for SNAP-25, PLXNA4 and UBA1.

Gene Name	Single marker	SNAP-25	PLXNA4	UBA1
SNAP-25	0.800	-	-	-
PLXNA4	0.763	-	-	-
UBA1	0.800	-	-	-
t-tau/EV (ELISA)	0.638	0.800	0.500	0.800
p-tau/EV (ELISA)	0.813	0.963	0.938	0.938

PLXNA4 is a member of a family of receptors for GPI-anchored semaphorins, including SEMA3A and SEMA6A [36]. Jun *et al.* have recently reported that PLXNA4 induces tau phosphorylation by activation of Cyclin-dependent kinase 5 and glycogen synthase kinase-3β, which are known to be activated by SEMA3A in Alzheimer’s disease [37,38], suggesting the potential implication of the same pathway in the p-tau accumulation in CTE brain, and its reflection in CTE EVs.

SNAP-25 is critical for synaptic vesicle fusion to the membrane and is implicated as a serum EV biomarker. The levels of SNAP-25 in serum neuron-derived EVs was reported to be reduced in AD patients [39]. Brinkmalm *et al*. have reported that the SNAP-25 fragment levels in CSF were significantly higher in AD compared to control cases [40]. We have previously identified the SNAP-25 in AD brain-derived EVs, and shown it was upregulated in AD patients compared to controls [22], suggesting this molecule as a common EV biomarker reflecting synaptic degeneration.

We also identified UBA1 in CTE EVs. UBA1 is the E1 ubiquitin-activating enzyme and represents an important regulator of cellular protein homeostasis. Specific disruption of UBA1 pathway is reported in in spinal muscular atrophy and Huntington's disease (HD) [41], which was also enriched in our IPA study. The expression level of UBA1 was significantly reduced in AD [42] and in HD brain tissues, and was negatively correlated with selective accumulation of toxic forms of huntingtin protein in the HD brains [41]. Thus, reduction of UBA1 in CTE brain tissue may be negatively correlated with enrichment of UBA1 in CTE EVs. The PLXNA4 and SNAP-25 have been known as neuron-specific markers. The combination of PLXNA4 or SNAP-25 and p-tau may be used to monitor the progression of CTE using body fluid sample. Our more recent study have not detected PLXNA4, SNAP-25 and UBA1 in the CSF EV samples from former NFL players [21]. Further study is necessary to validate these molecules using body fluid samples with higher sensitivity ELISA.

In summary, we have quantified of total and p-tau and profiled 516 common proteins in brain-derived EVs from CTE and control brain tissue samples. The combination of tau and cell type-specific molecules from brain cells, including PLXNA4, SNAP-25 or UBA1 may serve as potential monitoring biomarker candidate molecules in CTE patient body fluid samples.

## Supplementary Materials


