# Supplementary material for: Enrichment of Phosphorylated Tau (Thr181) and Functionally Interacting Molecules in Chronic Traumatic Encephalopathy Brain-derived Extracellular Vesicles
Source: Aging Dis. 2021 Sep 1;12(6):1376–88. doi: 10.14336/AD.2020.1007 (PMC8407888; doi:10.14336/AD.2020.1007)
Supplement: Supplementary file 1 [file AD-12-6-1376-s.pdf]

SUPPLEMENTARY DATA

Enrichment of Phosphorylated Tau (Thr181) and Functionally Interacting Molecules in Chronic Traumatic Encephalopathy Brain-derived Extracellular Vesicles

Satoshi Muraoka[1], Weiwei Lin[2,3], Kayo Takamatsu-Yukawa[1], Jianqiao Hu[1], Seiko Ikezu[1], Michael A. DeTure[4], Dennis W. Dickson[4], Andrew Emili[2,3], Tsuneya Ikezu[1,5,6*]

© 2020. Muraoka S et al. Published online at http://www.aginganddisease.org/EN/10.14336/AD.2020.1007

SUPPLEMENTARY DATA

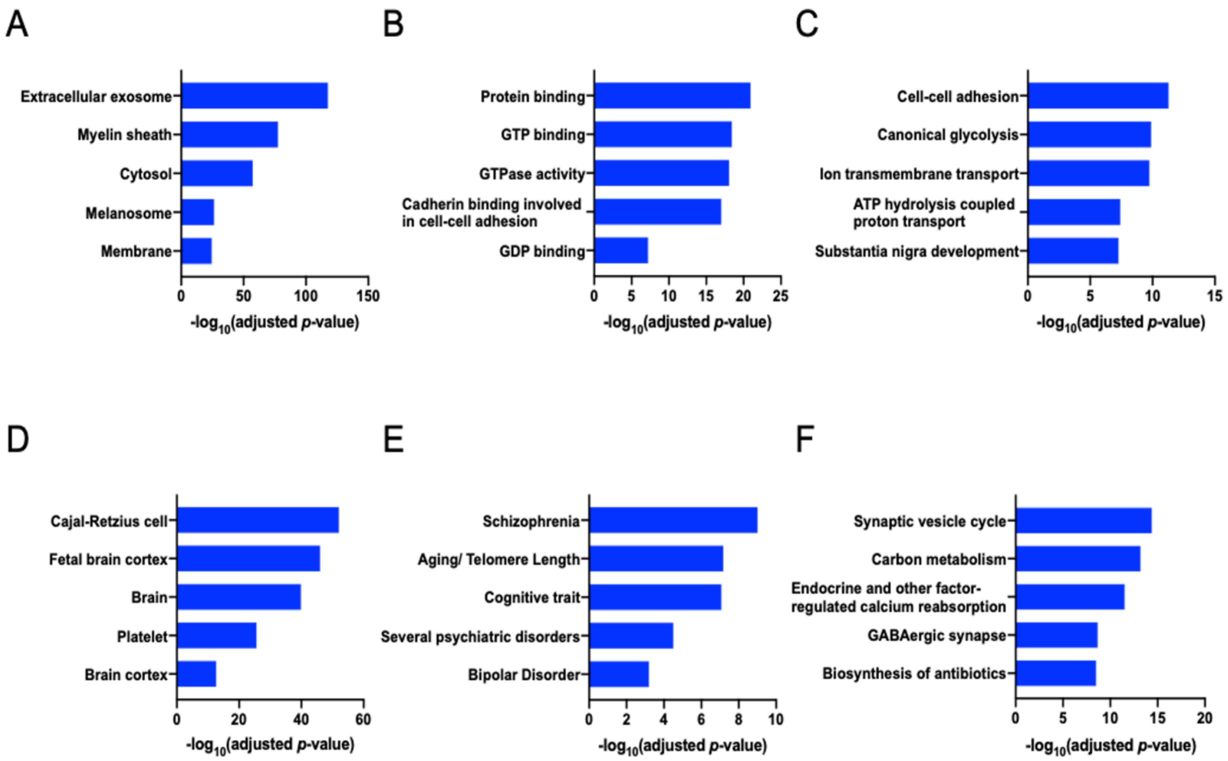

Supplementary Figure 1. Gene Ontology (GO) analysis of CTE brain-derived EV proteins using DAVID Bioinformatics Resources 6.8. The GO term of Top5 Cellular Component A), Molecular Function B), Biological process C), Tissue Expression Ontology D), Disease Ontology E), and KEGG Pathway F) with -log10(FDR p-value).

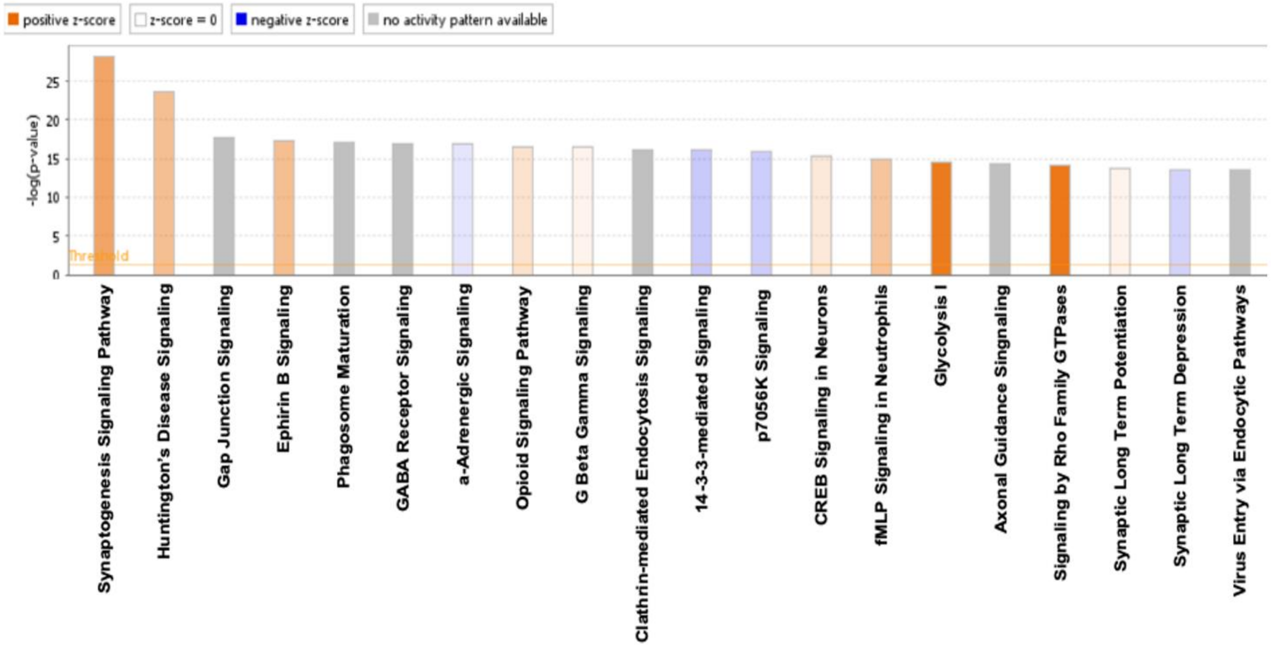

Supplementary Figure 2. Networks generated by Ingenuity pathway analysis. The canonical pathways that were up - and down - regulated in CTE compared to controls. Upregulated or down regulated pathway are denoted in red or blue.

© 2020. Muraoka S et al. Published online at http://www.aginganddisease.org/EN/10.14336/AD.2020.1007

SUPPLEMENTARY DATA

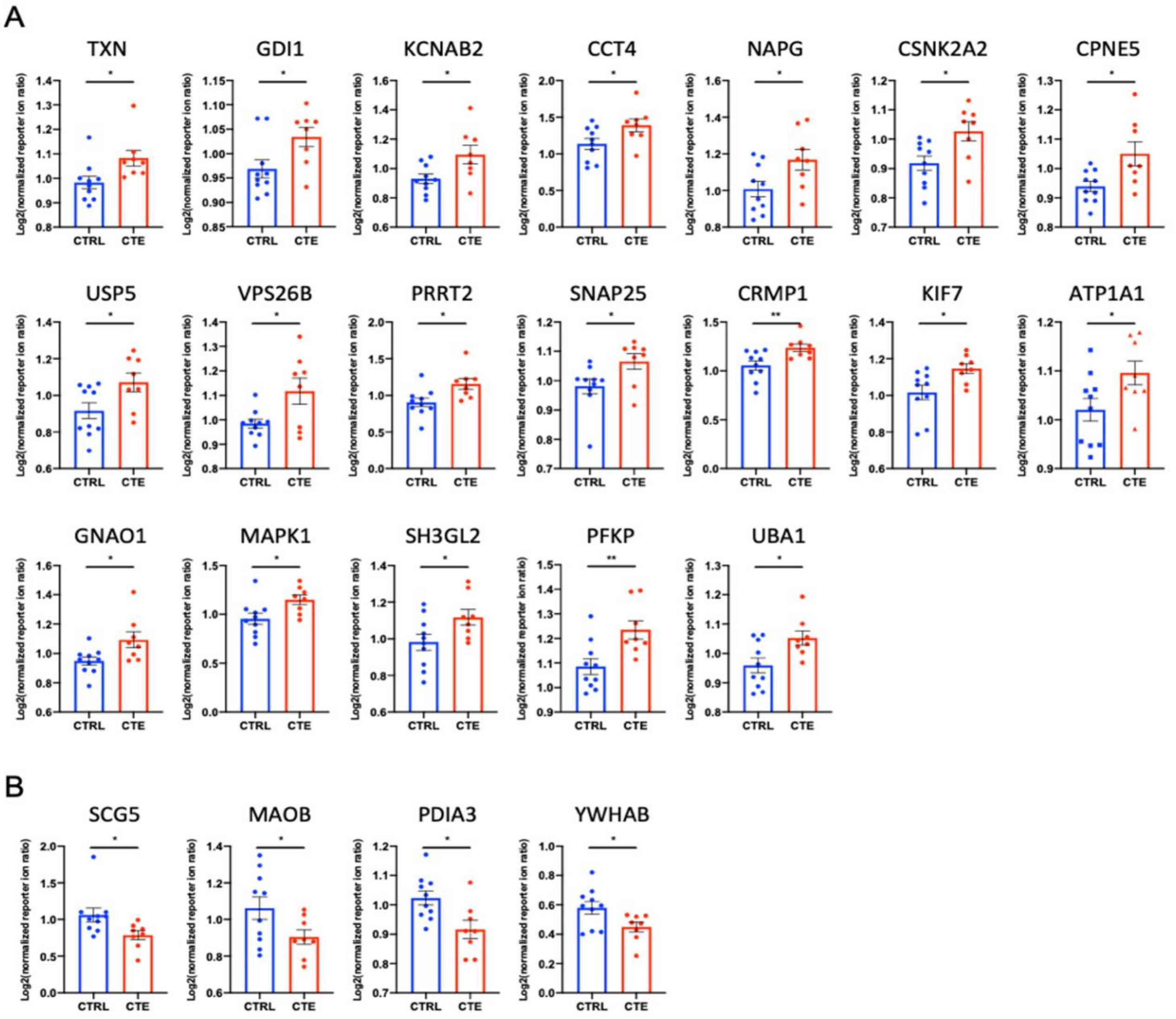

Supplementary Figure 3. A box plot of TMT-reporter intensity normalized by pooled sample as a standard control. The t-test was calculated by Welch's test. A) The 19 proteins were up-regulated in CTE compared to CTRL groups. **B)** The four proteins were down-regulated in CTE compared to CTRL groups.

© 2020. Muraoka S et al. Published online at http://www.aginganddisease.org/EN/10.14336/AD.2020.1007

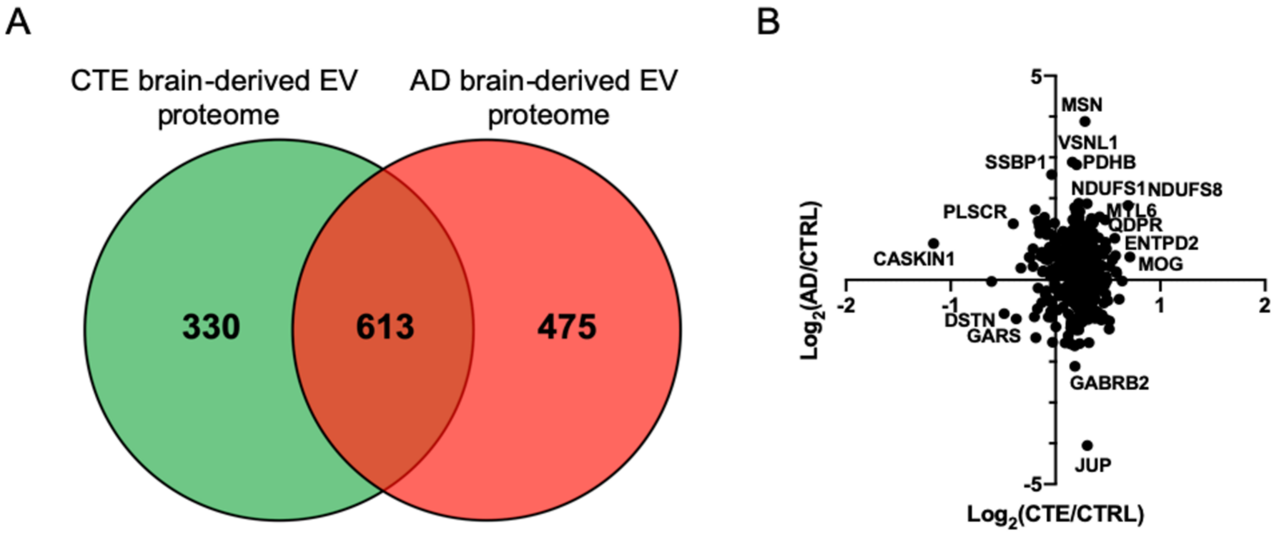

Supplementary Figure 4. Comparison of CTE brain -derived EV proteome and AD brain - derived EV proteome. A) The AD brain-derived EV proteome were identified 1080 proteins [1]. The 61 3 proteins were common between CTE brain -derived EV and AD brain -derived EV proteins. **B)** Scattered plot of CTE brain -derived EV and AD brain -derived EV proteins (r = - 0.0514, p = 0.2397 using two -tailed t -test).

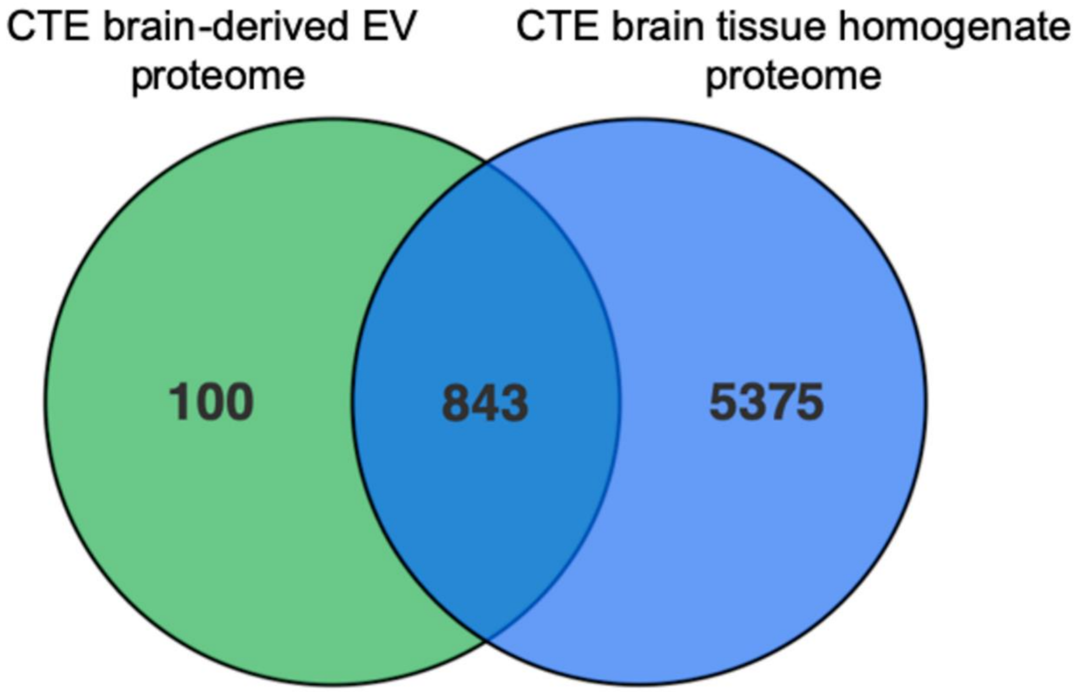

Supplementary Figure 5. Comparison of CTE brain-derived EV proteome and CTE brain tissue homogenates proteome. The CTE brain tissue homogenates proteomics data, which was published by Bi et al. was identified 6218 proteins [2]. The 843 proteins were common between CTE brain-derived EV and CTE brain tissue homogenate proteins.

© 2020. Muraoka S et al. Published online at http://www.aginganddisease.org/EN/10.14336/AD.2020.1007

SUPPLEMENTARY DATA

Supplementary Table 1. Assessment of the degree of purity of EV preparation.

	Transmembrane or GPI anchored proteins associated to plasma membrane and endosomes<br>For all EVs	
Category	Gene name	Uniprot ID
1a	GNAI2	P04899
	GNAO1	P09471
	GNAZ	P19086
	GNA11	P29992
	GNAQ	P50148
	GNAS	P63092
	GNAI1	P63096
	GNA13	Q14344
	HLA-B	P01889
	ITGAV	P06756
	ITGA6	P23229
	ITGB1	P05556
	EMMPRIN (BSG)	P35613
	ADAM10	O14672
	CD73 (NT5E)	P21589
	CD59	P13987
1b	CD90 (THY1)	P04216
	APP	P05067
	Cytosolic proteins recovered in EVs<br>For all EVs	
2a	ALIX (PDCD6IP)	Q8WUM4
	FLOT1	O75955
	FLOT2	Q14254
	EHD1	Q9H4M9
	EHD3	Q9NZN3
	EHD2	Q9NZN4
	RHOA	P61586
	ANXA1	P04083
	ANXA2	P07355
	ANXA6	P08133
	ANXA5	P08758

© 2020. Muraoka S et al. Published online at http://www.aginganddisease.org/EN/10.14336/AD.2020.1007

SUPPLEMENTARY DATA

	ANXA7	P20073
	ANXA11	P50995
	HSPA8	P11142
	HSP90AB1	P08238
	SDCBP	O00560
	Tau (MAPT)	P10636
2b	ACTG1	P63261
	TUBAL3	A6NHL2
	TPPP	O94811
	TUBB4A	P04350
	TUBB	P07437
	TUBA1A	Q71U36
	TUBA4A	P68366
	TUBB4B	P68371
	TUBB3	Q13509
	TUBB2B	Q9BVA1
	TUBB8	Q3ZCM7
	GAPDH	P04406
	Major components of non-EV co-isolated structures<br>For non-EV (contaminant)	
3a	ALB	P02768
	Analysis of protein of this category is required when claiming specific analysis of small EVs<br>Evs (large oncosomes, large EVs)<br>For small EVs	
4a (nucleus)	HIST1H2BL	Q99880
	HIST1H2AJ	Q99878
	HIST1H4A	P62805
4c (ER, Golgi)	Grp94 (HSP90B1)	P14625
	BIP (HSPA5)	P11021
4d (autophagosome)	ACTN1	P12814
	Analysis of protein of this category is required when claiming functional activities<br>functional activites<br>For functional component	
5b (adhesion)	MFGE8	Q08431

© 2020. Muraoka S et al. Published online at http://www.aginganddisease.org/EN/10.14336/AD.2020.1007

Tetraspanin	TSPAN7	P41732
ESCRT machinery	Rab4A	P20338
	Rab4B	P61018
	Rab5b	P61020
	Rab5c	P51148
	Rab11b	Q15907
	Rab7a	P51149
	VPS28	Q9UK41
	VPS36	Q86VN1
	Snf7-2(CHMP4B,VPS32B)	Q9H444
	Rab27b	O00194
	Rab35	Q15286
	syntenin-1(SDCBP)	O00560

Supplementary Table 2. Identification and quantification of EVs separated from CTE and controls brain tissue.

**Protein IDs**	**Gene names**	**Protein names**	Ave_CTRL	Ave_CTE	log_2(CTE/CTRL FC)	p-value[a]
P10599	TXN	Thioredoxin	0.982612065	1.082613861	0.139824906	0.03017023
Q01813	PFKP	ATP-dependent 6-phosphofructokinase, platelet type	1.085606193	1.235035538	0.186051699	0.00798276
P05408	SCG5	Neuroendocrine protein 7B2	1.062220295	0.785712598	-0.435009401	0.02676405
P19784	CSNK2A2	Casein kinase II subunit alpha	0.918166614	1.026720028	0.161214955	0.0177589
Q7Z6L0	PRRT2	Proline-rich transmembrane protein 2	0.904892386	1.155341546	0.352501273	0.01836626
Q14194	CRMP1	Dihydropyrimidinase-related protein 1	1.055683554	1.235843659	0.2273188	0.00948977
Q2M1P5	KIF7	Kinesin-like protein KIF7	1.015761905	1.145433765	0.173331764	0.0160733
P30101	PDIA3	Protein disulfide-isomerase A3	1.02330167	0.915955765	-0.159881684	0.0160897
P28482	MAPK1	Mitogen-activated protein kinase 1	0.954403551	1.147871841	0.26629026	0.02040801
P45974	USP5	Ubiquitin carboxyl-terminal hydrolase 5	0.916464824	1.071238358	0.225128112	0.03529199
P60880	SNAP25	Synaptosomal-associated protein 25	0.981106094	1.065253762	0.118716088	0.03648962
P09471	GNAO1	Guanine nucleotide-binding protein G(o) subunit alpha	0.948644336	1.09302717	0.204390062	0.03885183
Q13303	KCNAB2	Voltage-gated potassium channel subunit beta-2	0.930639255	1.094655054	0.234182375	0.0408079
Q9HCH3	CPNE5	Copine-5	0.939261395	1.050479096	0.161448835	0.03133789
P05023	ATP1A1	Sodium/potassium-transporting ATPase subunit alpha-1	1.020500163	1.095769097	0.10266741	0.04086187
P50991	CCT4	T-complex protein 1 subunit delta	1.135414227	1.389039333	0.290868728	0.04180259
P22314	UBA1	Ubiquitin-like modifier-activating enzyme 1	0.959177128	1.052764798	0.134313993	0.01668341
P31946	YWHAB	14-3-3 protein beta/alpha	0.579359454	0.448843351	-0.368246697	0.02909462

SUPPLEMENTARY DATA

Q99747	NAPG	Gamma-soluble NSF attachment protein	1.008103	1.16837079	0.212855146	0.03915817
Q6PIU2	NCEH1	Neutral cholesterol ester hydrolase 1	1.272657073	1.649581262	0.374256124	0.06161237
P09104	ENO2	Gamma-enolase	0.935193492	1.03838908	0.151010321	0.09073754
Q99962	SH3GL2	Endophilin-A1	0.981358666	1.116447738	0.186063307	0.04359349
Q09470	KCNA1	Potassium voltage-gated channel subfamily A member 1	0.98083712	1.117297332	0.187927678	0.06794857
P53396	ACLY	ATP-citrate synthase	1.038903882	1.088062233	0.066698893	0.09044608
Q96P70	IPO9	Importin-9	0.917206873	1.064370374	0.214681189	0.09865718
P23229	ITGA6	Integrin alpha-6	1.036748536	0.937558972	-0.145084666	0.10187327
Q9HCM2	PLXNA4	Plexin-A4	0.976464483	1.078772247	0.143750837	0.1171458
P31150	GDI1	Rab GDP dissociation inhibitor alpha	0.968822602	1.034160218	0.094155285	0.02791495
P27338	MAOB	Amine oxidase [flavin-containing] B	1.061473482	0.904472061	-0.230920487	0.04984126
Q13362	PPP2R5C	Serine/threonine-protein phosphatase 2A 56 kDa regulatory subunit gamma isoform	0.914288685	0.794451496	-0.202690625	0.05915672
Q9BSJ8	ESYT1	Extended synaptotagmin-1	1.006426227	1.122747054	0.157791514	0.06115764
O60716	CTNND1	Catenin delta-1	0.925113386	1.054007254	0.188182692	0.08825556
P15880	RPS2	40S ribosomal protein S2	1.230793478	1.069881313	-0.202137944	0.09545186
Q9Y376	CAB39	Calcium-binding protein 39	0.900883951	1.043823241	0.21246425	0.05279138
P49368	CCT3	T-complex protein 1 subunit gamma	0.981916027	1.091733561	0.152949251	0.06273029
P09417	QDPR	Dihydropteridine reductase	1.023330283	1.248824199	0.287298544	0.07424693
P16152	CBR1	Carbonyl reductase [NADPH] 1	1.04346246	1.138211357	0.125389781	0.07705886
P69905	HBA1	Hemoglobin subunit alpha	1.041767463	0.85982004	-0.276926642	0.08199897
Q01518	CAP1	Adenylyl cyclase-associated protein 1	1.046756571	1.1705398	0.161248014	0.09537515
O95248	SBF1	Myotubularin-related protein 5	1.07777102	1.195061915	0.149034664	0.09680421
P12277	CKB	Creatine kinase B-type	0.946818517	1.006695022	0.088466859	0.11105851
P30086	PEBP1	Phosphatidylethanolamine-binding protein 1	1.032470494	1.130739277	0.131165762	0.11139839
P28907	CD38	ADP-ribosyl cyclase/cyclic ADP-ribose hydrolase 1	1.075662815	1.213208895	0.17360207	0.11399442
P61266	STX1B	Syntaxin-1B	1.005389032	1.080905445	0.10448647	0.13824936
P63027	VAMP2	Vesicle-associated membrane protein 2	0.973884224	1.048020207	0.105844355	0.24635526
Q4G0F5	VPS26B	Vacuolar protein sorting-associated protein 26B	0.984501705	1.117332971	0.18259357	0.04534982
Q9BWD1	ACAT2	Acetyl-CoA acetyltransferase, cytosolic	0.817494164	1.001671045	0.293128459	0.10507972
Q96BM9	ARL8A	ADP-ribosylation factor-like protein 8A	1.087184702	1.02393186	-0.086477349	0.30395439
O75340	PDCD6	Programmed cell death protein 6	0.920825818	0.876526817	-0.071130053	0.35708879
Q16555	DPYSL2	Dihydropyrimidinase-related protein 2	0.910871991	0.991544663	0.122429439	0.36340199
P02794	FTH1	Ferritin heavy chain	0.448145549	0.3317706	-0.433781321	0.17193684
P10636	MAPT	Microtubule-associated protein tau	1.003851757	1.325968691	0.401500474	0.0683613
Q14515	SPARCL1	SPARC-like protein 1	0.980948044	0.839278509	-0.225027088	0.08457788
P06744	GPI	Glucose-6-phosphate isomerase	1.056095152	1.123245997	0.088934096	0.1103881
Q05193	DNM1	Dynamin-1	1.017043852	1.098685602	0.11139672	0.1112133
Q92598	HSPH1	Heat shock protein 105 kDa	1.015918343	1.145246733	0.172874001	0.1132252
O75110	ATP9A	Probable phospholipid-transporting ATPase IIA	0.956898483	0.824094438	-0.215556204	0.11877693

© 2020. Muraoka S et al. Published online at http://www.aginganddisease.org/EN/10.14336/AD.2020.1007

Q9Y2A7	NCKAP1	Nck-associated protein 1	0.938191141	1.01936322	0.119714422	0.12283608
Q86UW7	CADPS2	Calcium-dependent secretion activator 2	1.006201695	0.822699046	-0.290482848	0.17111361
P50914	RPL14	60S ribosomal protein L14	1.051315187	0.978907919	-0.102950193	0.18509263
Q13618	CUL3	Cullin-3	1.059081126	1.257564527	0.247819324	0.22308207
Q14203	DCTN1	Dynactin subunit 1	0.90548381	0.985034281	0.121485087	0.33072903
Q9P0J0	NDUFA13	NADH dehydrogenase [ubiquinone] 1 alpha subcomplex subunit 13	1.138406559	1.329197273	0.223539358	0.3615545
O43169	CYB5B	Cytochrome b5 type B	2.13416317	2.240950555	0.070440334	0.92542323
P17252	PRKCA	Protein kinase C alpha type	0.970330001	0.882697256	-0.136556766	0.08525173
A1L0T0	ILVBL	Acetolactate synthase-like protein	1.21368749	1.049905189	-0.20913794	0.09212492
P26232	CTNNA2	Catenin alpha-2	0.940720149	0.994102477	0.079628974	0.09730129
Q9Y2Q0	ATP8A1	Phospholipid-transporting ATPase IA	0.937532877	0.810189511	-0.210609874	0.14243824
O75781	PALM	Paralemmin-1	0.967723659	1.076066824	0.153100634	0.14884672
P14174	MIF	Macrophage migration inhibitory factor	1.043939213	1.117030354	0.097630682	0.22649306
P84085	ARF5	ADP-ribosylation factor 5	0.906290002	0.846937186	-0.097717795	0.23177794
Q9NS86	LANCL2	LanC-like protein 2	1.148146464	1.020797272	-0.169610313	0.25241429
Q9NP79	VTA1	Vacuolar protein sorting-associated protein VTA1 homolog	0.951069703	0.879259951	-0.113261322	0.27366587
P08237	PFKM	ATP-dependent 6-phosphofructokinase, muscle type	1.017346303	1.052810635	0.049435113	0.34447106
Q13424	SNTA1	Alpha-1-syntrophin	1.02142877	0.978266167	-0.062289648	0.58667377
Q86Y82	STX12	Syntaxin-12	1.107764005	1.016167589	-0.124512211	0.06938293
Q9Y4I1	MYO5A	Unconventional myosin-Va	0.95545354	1.042540638	0.125845994	0.07436188
P48147	PREP	Prolyl endopeptidase	0.903409501	1.008231481	0.158374914	0.08746098
P04271	S100B	Protein S100-B	0.776861888	0.70328252	-0.143553777	0.1033322
P49720	PSMB3	Proteasome subunit beta type-3	0.906371267	1.024075256	0.176147707	0.1057368
P17600	SYN1	Synapsin-1	0.967258858	1.044953413	0.111464684	0.11402851
Q99798	ACO2	Aconitate hydratase, mitochondrial	1.025601632	0.960350762	-0.09483712	0.14427487
O43175	PHGDH	D-3-phosphoglycerate dehydrogenase	0.968996428	1.158746398	0.258001602	0.16430484
P40925	MDH1	Malate dehydrogenase, cytoplasmic	0.942095378	1.025272155	0.122061889	0.16507586
P63261	ACTG1	Actin	0.943728435	1.007367774	0.094146806	0.18596046
O00410	IPO5	Importin-5	1.024037665	1.123391911	0.133592541	0.21056338
Q8NEV1	CSNK2A3	Casein kinase II subunit alpha 3	0.869727732	0.922852806	0.085536722	0.21904509
P55786	NPEPPS	Puromycin-sensitive aminopeptidase	0.9585094	1.030714727	0.104780606	0.25276125
Q9UQM7	CAMK2A	Calcium/calmodulin-dependent protein kinase type II subunit alpha	0.970873153	1.023301551	0.075876626	0.26358508
Q9NZN3	EHD3	EH domain-containing protein 3	0.962597273	1.005565898	0.063003388	0.27032001
O75915	ARL6IP5	PRA1 family protein 3	0.976972165	0.925079202	-0.078740568	0.27045455
P07195	LDHB	L-lactate dehydrogenase B chain	1.065088813	1.199987405	0.172045527	0.36049196
P63000	RAC1	Ras-related C3 botulinum toxin substrate 1	1.068980274	1.201492084	0.168591912	0.39519438
P05026	ATP1B1	Sodium/potassium-transporting ATPase subunit beta-1	0.988942486	0.894079416	-0.145483638	0.44743964
P09382	LGALS1	Galectin-1	1.063676163	1.004136494	-0.083103598	0.54447377
O00483	NDUFA4	Cytochrome c oxidase subunit NDUFA4	1.009674672	0.963974296	-0.066823932	0.69538087

© 2020. Muraoka S et al. Published online at http://www.aginganddisease.org/EN/10.14336/AD.2020.1007

SUPPLEMENTARY DATA

O60268	KIAA0513	Uncharacterized protein KIAA0513	1.10507927	1.212320134	0.133620856	0.1015544
Q7Z3U7	MON2	Protein MON2 homolog	1.009095705	0.864483976	-0.223151882	0.12748011
Q15041	ARL6IP1	ADP-ribosylation factor-like protein 6-interacting protein 1	1.045849723	0.878842092	-0.250999693	0.13160171
P00533	EGFR	Epidermal growth factor receptor	1.008166123	1.136940565	0.173423456	0.13194395
Q9UBQ0	VPS29	Vacuolar protein sorting-associated protein 29	1.132090478	1.366122069	0.271097136	0.15286866
A6NHL2	TUBAL3	Tubulin alpha chain-like 3	1.06315508	1.236560056	0.217980253	0.16816353
Q96GG9	DCUN1D1	DCN1-like protein 1	1.052848964	0.936667531	-0.168689529	0.18367177
P04264	KRT1	Keratin, type II cytoskeletal 1	0.967086869	1.020377245	0.077385241	0.18741636
O43301	HSPA12A	Heat shock 70 kDa protein 12A	0.978804694	1.012403705	0.048691767	0.20479927
P60842	EIF4A1	Eukaryotic initiation factor 4A-I	0.920698886	0.952478126	0.048956559	0.20801122
Q9NZ32	ACTR10	Actin-related protein 10	1.010337044	0.92318905	-0.130138633	0.23345782
Q9ULP9	TBC1D24	TBC1 domain family member 24	0.945830879	1.050897624	0.151967984	0.2433588
P21283	ATP6V1C1	V-type proton ATPase subunit C 1	1.08091819	0.934221355	-0.210421008	0.24516676
P04350	TUBB4A	Tubulin beta-4A chain	0.950169562	1.003394736	0.078632377	0.27197948
Q96CS3	FAF2	FAS-associated factor 2	1.102912551	1.179494886	0.096850758	0.27388793
P29401	TKT	Transketolase	0.96871469	1.026358369	0.083390834	0.28544996
Q15102	PAFAH1B3	Platelet-activating factor acetylhydrolase IB subunit gamma	1.06372751	1.139071634	0.098729847	0.29555027
Q96JE9	MAP6	Microtubule-associated protein 6	1.039625288	1.130291427	0.120631164	0.31691167
P61604	HSPE1	10 kDa heat shock protein, mitochondrial	1.088189971	0.949779795	-0.196265467	0.32533134
Q9BY11	PACSIN1	Protein kinase C and casein kinase substrate in neurons protein 1	1.008453522	1.054356561	0.064218245	0.36144783
Q14254	FLOT2	Flotillin-2	0.886905268	0.948704749	0.097179154	0.3659768
P23634	ATP2B4	Plasma membrane calcium-transporting ATPase 4	1.056499538	1.118106339	0.081765267	0.37063832
P49327	FASN	Fatty acid synthase	1.038849577	1.134146444	0.126620167	0.43807416
O14807	MRAS	Ras-related protein M-Ras	0.960028369	1.031829452	0.104055588	0.55636199
O43426	SYNJ1	Synaptojanin-1	1.078981344	0.956499666	-0.173833551	0.14135747
Q9GZN7	ROGDI	Protein rogdi homolog	0.899988277	0.990762871	0.138633595	0.14620629
P22695	UQCRC2	Cytochrome b-c1 complex subunit 2, mitochondrial	1.083186817	1.008079788	-0.103672255	0.21223858
Q13363	CTBP1	C-terminal-binding protein 1	0.949848538	0.880587258	-0.109231511	0.22483683
P00568	AK1	Adenylate kinase isoenzyme 1	1.031381874	0.967212395	-0.092673959	0.2258126
Q02818	NUCB1	Nucleobindin-1	0.921189388	0.847688911	-0.119962877	0.24823236
P78352	DLG4	Disks large homolog 4	0.960874935	1.197681478	0.317823706	0.26082387
P19367	HK1	Hexokinase-1	1.082296633	1.008887352	-0.101330865	0.26160237
P07437	TUBB	Tubulin beta chain	0.90085857	0.947258656	0.07245779	0.26635224
Q92597	NDRG1	Protein NDRG1	1.055256387	1.185801198	0.168268598	0.26654388
P60891	PRPS1	Ribose-phosphate pyrophosphokinase 1	0.942363461	0.987890988	0.068068251	0.28672437
Q04837	SSBP1	Single-stranded DNA-binding protein, mitochondrial	1.179058055	0.98540511	-0.258845898	0.29628889
P61421	ATP6V0D1	V-type proton ATPase subunit d 1	1.049594628	0.984507748	-0.092357776	0.32718953
O75390	CS	Citrate synthase, mitochondrial	1.015032267	0.91614844	-0.147872313	0.34083914
P50148	GNAQ	Guanine nucleotide-binding protein G(q) subunit alpha	0.994836159	0.97272151	-0.032432125	0.35508835

SUPPLEMENTARY DATA

Q9UBB6	NCDN	Neurochondrin	0.948435753	1.016773156	0.100375892	0.36138462
O00154	ACOT7	Cytosolic acyl coenzyme A thioester hydrolase	0.981876342	0.925802849	-0.084836339	0.37512237
P11021	HSPA5	78 kDa glucose-regulated protein	1.096986762	1.06084861	-0.048327326	0.55224751
Q9Y2S2	CRYL1	Lambda-crystallin homolog	0.871894902	0.84517934	-0.044896742	0.56567216
P16298	PPP3CB	Serine/threonine-protein phosphatase 2B catalytic subunit beta isoform	0.896143137	0.905545698	0.015058262	0.83186589
P42262	GRIA2	Glutamate receptor 2	1.068507713	0.979317936	-0.125748109	0.10380652
P55290	CDH13	Cadherin-13	0.979055581	1.0457596	0.095088572	0.19380398
Q93009	USP7	Ubiquitin carboxyl-terminal hydrolase 7	0.938792714	1.017633164	0.116339042	0.19962389
P18124	RPL7	60S ribosomal protein L7	1.07982596	0.955350961	-0.176696078	0.21645615
P08133	ANXA6	Annexin A6	0.951975329	0.981310443	0.043785427	0.22873287
Q13098	GPS1	COP9 signalosome complex subunit 1	0.906389945	1.005002857	0.14899584	0.23447074
O00764	PDXK	Pyridoxal kinase	0.955383757	1.013953065	0.085838619	0.29753879
P55072	VCP	Transitional endoplasmic reticulum ATPase	1.098564768	1.171998003	0.093350183	0.31002739
Q99584	S100A13	Protein S100-A13	0.6458724	0.889164724	0.461201543	0.31135091
A6NDG6	PGP	Phosphoglycolate phosphatase	1.016118748	1.1120328	0.130130331	0.32755855
P20338	RAB4A	Ras-related protein Rab-4A	0.992020117	1.060604767	0.096445857	0.36090006
Q15165	PON2	Serum paraoxonase/arylesterase 2	1.072468489	0.963133226	-0.155127982	0.37563003
P22626	HNRNPA2B1	Heterogeneous nuclear ribonucleoproteins A2/B1	0.842950473	0.779692451	-0.112542703	0.40574879
Q15904	ATP6AP1	V-type proton ATPase subunit S1	1.091338869	1.159369265	0.087241006	0.42581912
P61019	RAB2A	Ras-related protein Rab-2A	0.924466387	0.903074759	-0.033775443	0.45156001
P14625	HSP90B1	Endoplasmin	1.062987138	1.009284283	-0.074791547	0.56646411
Q9NYB9	ABI2	Abl interactor 2	1.064803692	1.099340266	0.046050517	0.61665665
Q9H4M9	EHD1	EH domain-containing protein 1	1.049537017	1.066177476	0.022694558	0.69795044
Q16658	FSCN1	Fascin	0.989422084	0.994229614	0.006992975	0.92017209
P01112	HRAS	GTPase HRas	0.980252166	0.977013524	-0.004774392	0.96682219
P62873	GNB1	Guanine nucleotide-binding protein G(I)/G(S)/G(T) subunit beta-1	0.934628809	0.935621051	0.001530814	0.97637669
Q15907	RAB11B	Ras-related protein Rab-11B	1.009831985	1.109818334	0.136208263	0.20605488
Q9Y639	NPTN	Neuroplastin	1.001427737	0.924064046	-0.115993568	0.2376465
Q9UI12	ATP6V1H	V-type proton ATPase subunit H	1.000446187	1.065618943	0.091048066	0.2416132
P13987	CD59	CD59 glycoprotein	1.164275237	1.032499586	-0.17329095	0.27252929
Q02252	ALDH6A1	Methylmalonate-semialdehyde dehydrogenase [acylating], mitochondrial	0.949408806	1.060673489	0.159879279	0.27711895
Q969P0	IGSF8	Immunoglobulin superfamily member 8	1.035068381	1.081369999	0.063134156	0.31465542
Q92777	SYN2	Synapsin-2	1.058763213	1.141862204	0.109008588	0.32325125
Q68D91	MBLAC2	Metallo-beta-lactamase domain-containing protein 2	1.004608816	1.067225096	0.087230656	0.34033534
Q9UMF0	ICAM5	Intercellular adhesion molecule 5	1.039406458	0.980400148	-0.084317321	0.37079596
Q14974	KPNB1	Importin subunit beta-1	1.132658096	1.231479951	0.120680706	0.37812073
Q96QK1	VPS35	Vacuolar protein sorting-associated protein 35	1.004274614	1.060373721	0.078419001	0.41237356
P30153	PPP2R1A	Serine/threonine-protein phosphatase 2A 65 kDa regulatory subunit A alpha isoform	1.004270969	1.047282576	0.060502175	0.43583035
O43681	ASNA1	ATPase ASNA1	0.967661888	0.99859535	0.045397147	0.45223168

P36543	ATP6V1E1	V-type proton ATPase subunit E 1	0.979193465	1.044856878	0.093639504	0.45905423
P06307	CCK	Cholecystokinin	0.945642365	1.002434054	0.084140756	0.47454806
Q00169	PITPNA	Phosphatidylinositol transfer protein alpha isoform	0.961544989	0.906658997	-0.084794319	0.49040622
P00338	LDHA	L-lactate dehydrogenase A chain	1.002538966	1.057139753	0.076507801	0.50792775
O15145	ARPC3	Actin-related protein 2/3 complex subunit 3	0.904668054	0.93537508	0.048156465	0.65199394
P14867	GABRA1	Gamma-aminobutyric acid receptor subunit alpha-1	1.075324764	1.104784871	0.038993027	0.6706987
P02511	CRYAB	Alpha-crystallin B chain	0.992763569	0.981354651	-0.016675569	0.74562418
P40926	MDH2	Malate dehydrogenase, mitochondrial	1.125728337	1.15043926	0.031326099	0.91394278
Q9UI15	TAGLN3	Transgelin-3	0.989068773	1.092579991	0.143596164	0.24885081
O75306	NDUFS2	NADH dehydrogenase [ubiquinone] iron-sulfur protein 2, mitochondrial	1.009081191	0.873070612	-0.208872014	0.27176613
O94973	AP2A2	AP-2 complex subunit alpha-2	0.97885472	1.04475526	0.093998364	0.31642722
P09936	UCHL1	Ubiquitin carboxyl-terminal hydrolase isozyme L1	1.021488333	1.098110274	0.104350213	0.33481606
Q99726	SLC30A3	Zinc transporter 3	1.093242001	0.980676695	-0.156763294	0.36369731
Q00610	CLTC	Clathrin heavy chain 1	0.940812436	0.973041608	0.048594367	0.38505677
P25705	ATP5A1	ATP synthase subunit alpha, mitochondrial	0.937375411	0.889773907	-0.075188159	0.39437362
P28331	NDUFS1	NADH-ubiquinone oxidoreductase 75 kDa subunit, mitochondrial	1.038732187	1.109448311	0.095018718	0.40660574
P30626	SRI	Sorcin	1.150601438	1.253449238	0.123515394	0.41898102
P11234	RALB	Ras-related protein Ral-B	0.915068856	0.843421702	-0.117626162	0.42193721
P62879	GNB2	Guanine nucleotide-binding protein G(I)/G(S)/G(T) subunit beta-2	0.983512541	1.013009919	0.042632946	0.42994396
P18669	PGAM1	Phosphoglycerate mutase 1	0.823186131	0.741772538	-0.150241819	0.44530493
Q9H4G0	EPB41L1	Band 4.1-like protein 1	1.090572983	1.13168407	0.053384939	0.5269328
P54920	NAPA	Alpha-soluble NSF attachment protein	0.98052614	0.935752876	-0.067428515	0.56524083
Q8IV08	PLD3	Phospholipase D3	1.090965671	1.073146052	-0.02375927	0.6862825
Q14195	DPYSL3	Dihydropyrimidinase-related protein 3	1.165931701	1.191346192	0.031109426	0.82900167
Q16775	HAGH	Hydroxyacylglutathione hydrolase, mitochondrial	0.934533968	1.099600061	0.234659884	0.21458832
P34932	HSPA4	Heat shock 70 kDa protein 4	0.987829215	0.925242389	-0.094430274	0.21733314
P38606	ATP6V1A	V-type proton ATPase catalytic subunit A	0.992606478	1.035235146	0.060664727	0.22177073
P47755	CAPZA2	F-actin-capping protein subunit alpha-2	1.02801484	1.119073588	0.122443818	0.24712946
P99999	CYCS	Cytochrome c	0.89444553	0.826668266	-0.113685122	0.30233051
Q9H0U4	RAB1B	Ras-related protein Rab-1B	0.945120462	0.993153927	0.071519114	0.31475079
P04899	GNAI2	Guanine nucleotide-binding protein G(i) subunit alpha-2	1.051655047	1.185298649	0.172589043	0.33557953
P13611	VCAN	Versican core protein	0.974697232	1.038107932	0.090930396	0.33705854
O75955	FLOT1	Flotillin-1	1.013383963	1.123397853	0.148688047	0.35835031
P62195	PSMC5	26S protease regulatory subunit 8	0.908257979	0.982350721	0.113136056	0.36305189
P32418	SLC8A1	Sodium/calcium exchanger 1	1.108430231	1.175848325	0.085184013	0.36501147
Q07960	ARHGAP1	Rho GTPase-activating protein 1	0.924771696	0.98473306	0.090635451	0.36655034
Q15435	PPP1R7	Protein phosphatase 1 regulatory subunit 7	1.025136062	1.060249956	0.048589018	0.38661739
Q9H310	RHBG	Ammonium transporter Rh type B	0.975917126	0.928796303	-0.071396411	0.42309181
P11216	PYGB	Glycogen phosphorylase, brain form	0.980069278	1.004690174	0.035795035	0.42637202

P67936	TPM4	Tropomyosin alpha-4 chain	0.974545662	1.014051857	0.057329742	0.42864394
Q9BTV4	TMEM43	Transmembrane protein 43	0.811647486	0.881259848	0.118714201	0.43256011
P06733	ENO1	Alpha-enolase	0.99820772	1.038512826	0.057107067	0.43340158
Q5TF21	SOGA3	Protein SOGA3	1.294982056	1.469052338	0.181953688	0.46284987
O15484	CAPN5	Calpain-5	0.870431427	0.899323366	0.047109306	0.51268297
P13489	RNH1	Ribonuclease inhibitor	0.929891782	0.889765914	-0.063636999	0.54300615
Q9UHY7	ENOPH1	Enolase-phosphatase E1	0.978369157	0.950309144	-0.041982013	0.60934112
P04075	ALDOA	Fructose-bisphosphate aldolase A	1.019138455	0.997827021	-0.030488418	0.64241383
Q9UM19	HPCAL4	Hippocalcin-like protein 4	0.839685212	0.823934451	-0.027319012	0.65052604
Q9UQB3	CTNND2	Catenin delta-2	0.868997718	0.848509374	-0.03442179	0.65755074
Q9UPR5	SLC8A2	Sodium/calcium exchanger 2	0.924714041	0.949806497	0.038626329	0.66826856
Q9ULX5	RNF112	RING finger protein 112	0.814440483	0.861545206	0.081117222	0.66860068
P62826	RAN	GTP-binding nuclear protein Ran	1.013278239	1.060360988	0.065525115	0.67289759
O43581	SYT7	Synaptotagmin-7	0.830915867	0.873565429	0.072213358	0.67786183
P20073	ANXA7	Annexin A7	1.04515621	1.063852556	0.02557963	0.68930663
P09972	ALDOC	Fructose-bisphosphate aldolase C	1.022944023	1.036230975	0.018618414	0.77697628
P61106	RAB14	Ras-related protein Rab-14	0.924079309	0.90855531	-0.024442331	0.81671628
P14927	UQCRB	Cytochrome b-c1 complex subunit 7	1.257935813	1.267751791	0.011214003	0.93243293
P63104	YWHAZ	14-3-3 protein zeta/delta	0.964347175	0.966711452	0.003532707	0.96569064
P08758	ANXA5	Annexin A5	1.03930865	0.912790609	-0.18726831	0.24028784
Q01814	ATP2B2	Plasma membrane calcium-transporting ATPase 2	1.015809141	1.088256274	0.099388976	0.25709612
P68366	TUBA4A	Tubulin alpha-4A chain	0.895734589	0.943646518	0.075175222	0.26062854
Q06124	PTPN11	Tyrosine-protein phosphatase non-receptor type 11	1.007122439	1.123242591	0.15743046	0.28418039
P63010	AP2B1	AP-2 complex subunit beta	0.966812525	0.93957615	-0.04122607	0.30118835
Q9NRW1	RAB6B	Ras-related protein Rab-6B	0.948629676	1.004940262	0.083192838	0.36835636
P10768	ESD	S-formylglutathione hydrolase	1.006953946	1.061705584	0.076386053	0.37611532
Q9NQ66	PLCB1	1-phosphatidylinositol 4,5-bisphosphate phosphodiesterase beta-1	1.046102365	1.104516342	0.078390733	0.40973947
Q9ULU8	CADPS	Calcium-dependent secretion activator 1	0.940692587	0.98183518	0.061757526	0.41237438
P21266	GSTM3	Glutathione S-transferase Mu 3	0.977636747	1.050755405	0.104056458	0.42645556
P05091	ALDH2	Aldehyde dehydrogenase, mitochondrial	1.074683731	1.039121866	-0.048547291	0.45732017
P48163	ME1	NADP-dependent malic enzyme	1.054212774	0.989240389	-0.091773029	0.479088
P15311	EZR	Ezrin	0.978627473	0.949779777	-0.043166745	0.52798404
Q7Z6G3	NECAB2	N-terminal EF-hand calcium-binding protein 2	1.009737174	1.050308795	0.056833729	0.53854102
O60331	PIP5K1C	Phosphatidylinositol 4-phosphate 5-kinase type-1 gamma	1.112444476	1.143542456	0.039776599	0.55611696
P54289	CACNA2D1	Voltage-dependent calcium channel subunit alpha-2/delta-1	0.964302173	0.924255916	-0.061192926	0.56102103
Q8NCB2	CAMKV	CaM kinase-like vesicle-associated protein	1.013771525	1.045452462	0.044394916	0.56131346
Q12860	CNTN1	Contactin-1	0.993146908	0.943603782	-0.073825939	0.57380665
P07900	HSP90AA1	Heat shock protein HSP 90-alpha	0.968066868	0.99476878	0.039254527	0.59467619
O60641	SNAP91	Clathrin coat assembly protein AP180	0.73926808	0.824756349	0.157870356	0.60386309

© 2020. Muraoka S et al. Published online at http://www.aginganddisease.org/EN/10.14336/AD.2020.1007

P29218	IMPA1	Inositol monophosphatase 1	1.013256326	0.985691932	-0.03979046	0.644785
Q15293	RCN1	Reticulocalbin-1	0.999113874	0.974315326	-0.036260359	0.68490195
P63096	GNAI1	Guanine nucleotide-binding protein G(i) subunit alpha-1	0.824243486	0.851300039	0.046597115	0.7441918
O00533	CHL1	Neural cell adhesion molecule L1-like protein	1.127638455	1.098414159	-0.037882456	0.7619545
P18505	GABRB1	Gamma-aminobutyric acid receptor subunit beta-1	1.051258716	1.036389744	-0.020551117	0.7905148
O60675	MAFK	Transcription factor MafK	1.120955325	1.109139736	-0.015287645	0.938752
P56134	ATP5J2	ATP synthase subunit f, mitochondrial	1.054582398	0.906027779	-0.219044633	0.29643576
Q7L099	RUFY3	Protein RUFY3	1.007708686	1.081881451	0.102463785	0.31395863
O14531	DPYSL4	Dihydropyrimidinase-related protein 4	1.144671132	1.279989717	0.161199054	0.31818314
P28472	GABRB3	Gamma-aminobutyric acid receptor subunit beta-3	1.080940046	0.992936244	-0.122513516	0.33208451
Q14416	GRM2	Metabotropic glutamate receptor 2	0.982950543	1.131608413	0.203184073	0.3477447
P13637	ATP1A3	Sodium/potassium-transporting ATPase subunit alpha-3	1.019379219	1.061378595	0.058248511	0.35284286
O75935	DCTN3	Dynactin subunit 3	1.091329041	1.191244151	0.126382984	0.35514975
Q71U36	TUBA1A	Tubulin alpha-1A chain	0.850296406	0.942193358	0.148057322	0.38510228
Q05639	EEF1A2	Elongation factor 1-alpha 2	0.999168965	0.961390687	-0.055605838	0.39151036
P35611	ADD1	Alpha-adducin	1.019064467	0.985397163	-0.048468099	0.40041465
O95336	PGLS	6-phosphogluconolactonase	0.982855309	1.044087644	0.087191871	0.40518037
Q9NV96	TMEM30A	Cell cycle control protein 50A	1.213346224	1.129378222	-0.10346256	0.43387329
P08559	PDHA1	Pyruvate dehydrogenase E1 component subunit alpha, somatic form, mitochondrial	1.072963199	1.007642699	-0.090616431	0.43906596
P14618	PKM	Pyruvate kinase PKM	0.960259815	0.9978866	0.055451072	0.45262136
P52306	RAP1GDS1	Rap1 GTPase-GDP dissociation stimulator 1	0.89617333	0.825337585	-0.118793452	0.46365255
Q9Y5K8	ATP6V1D	V-type proton ATPase subunit D	1.082684824	1.150986213	0.088257226	0.47503975
P20020	ATP2B1	Plasma membrane calcium-transporting ATPase 1	0.915296617	0.954414402	0.060376465	0.49373863
O00429	DNM1L	Dynamin-1-like protein	0.961251902	0.985973961	0.036634999	0.49443703
Q13367	AP3B2	AP-3 complex subunit beta-2	0.916066309	0.955853607	0.06133765	0.51066726
P36915	GNL1	Guanine nucleotide-binding protein-like 1	0.964288328	1.019158991	0.079842643	0.51186582
P21579	SYT1	Synaptotagmin-1	0.974276183	0.950612774	-0.03547301	0.51637687
P53680	AP2S1	AP-2 complex subunit sigma	1.032645787	1.067191194	0.047473195	0.59043012
P09543	CNP	2,3-cyclic-nucleotide 3-phosphodiesterase	1.016460548	0.977928469	-0.055753374	0.60269622
P05186	ALPL	Alkaline phosphatase, tissue-nonspecific isozyme	1.029721628	1.071336493	0.057157307	0.60566525
Q16348	SLC15A2	Solute carrier family 15 member 2	0.986415479	0.945796544	-0.060665569	0.63430599
O94811	TPPP	Tubulin polymerization-promoting protein	0.976087947	1.002082967	0.037918913	0.63737977
P78356	PIP4K2B	Phosphatidylinositol 5-phosphate 4-kinase type-2 beta	0.95421541	0.986519523	0.048032617	0.6853027
Q07954	LRP1	Prolow-density lipoprotein receptor-related protein 1	1.049626453	1.011958414	-0.052725981	0.69920957
O14576	DYNC1I1	Cytoplasmic dynein 1 intermediate chain 1	0.779951023	0.738216104	-0.079340322	0.7105416
P32119	PRDX2	Peroxiredoxin-2	1.004467875	1.025504516	0.029902421	0.71538862
P14136	GFAP	Glial fibrillary acidic protein	0.952405631	0.934250544	-0.027766652	0.77670186
P14406	COX7A2	Cytochrome c oxidase subunit 7A2, mitochondrial	1.074166439	1.051756955	-0.030416194	0.81335978

© 2020. Muraoka S et al. Published online at http://www.aginganddisease.org/EN/10.14336/AD.2020.1007

O14773	TPP1	Tripeptidyl-peptidase 1	1.107076431	1.115849152	0.01138718	0.88383884
Q9NQR4	NIT2	Omega-amidase NIT2	0.86976434	0.860238808	-0.015887343	0.88487127
Q8N608	DPP10	Inactive dipeptidyl peptidase 10	0.989626906	1.003383732	0.019916822	0.905885
Q06732	ZNF33B	Zinc finger protein 33B	1.164443034	1.189408382	0.030604086	0.91341812
P11217	PYGM	Glycogen phosphorylase, muscle form	1.041652114	1.037434915	-0.005852703	0.94779061
P05067	APP	Amyloid beta A4 protein	1.122867851	1.113694909	-0.011834081	0.95245834
Q15008	PSMD6	26S proteasome non-ATPase regulatory subunit 6	1.022462811	1.027520278	0.007118495	0.95583479
Q9UPV7	KIAA1045	Protein KIAA1045	0.956361807	0.957815206	0.002190824	0.98576276
P30531	SLC6A1	Sodium- and chloride-dependent GABA transporter 1	0.872402576	1.06142736	0.282939707	0.2540137
Q13200	PSMD2	26S proteasome non-ATPase regulatory subunit 2	0.970004558	1.026287446	0.081371431	0.27579337
P60174	TPI1	Triosephosphate isomerase	0.919803266	1.061876617	0.20721892	0.32252493
Q99880	HIST1H2BL	Histone H2B type 1-L	0.966033672	1.035788437	0.100583976	0.38628255
P12532	CKMT1A	Creatine kinase U-type, mitochondrial	1.01082686	1.072682847	0.085687681	0.40876001
Q14204	DYNC1H1	Cytoplasmic dynein 1 heavy chain 1	0.978198527	1.012021004	0.049040036	0.41877956
P17174	GOT1	Aspartate aminotransferase, cytoplasmic	1.289030011	1.09646624	-0.23342446	0.47032646
Q13561	DCTN2	Dynactin subunit 2	0.995030054	0.95730044	-0.055768329	0.51344237
Q8WUW1	BRK1	Protein BRICK1	1.055724289	1.077043642	0.028843597	0.53366109
Q92752	TNR	Tenascin-R	1.007799191	0.958769326	-0.071952544	0.54744707
P63151	PPP2R2A	Serine/threonine-protein phosphatase 2A 55 kDa regulatory subunit B alpha isoform	0.944883718	0.991342002	0.069246063	0.5981944
Q8WUM4	PDCD6IP	Programmed cell death 6-interacting protein	0.916261065	0.889249984	-0.043169672	0.61716277
P10909	CLU	Clusterin	0.981728166	0.939745204	-0.06305396	0.61762964
Q13554	CAMK2B	Calcium/calmodulin-dependent protein kinase type II subunit beta	0.932571744	0.952355568	0.030285596	0.6440191
Q92599	SEP8	Septin-8	1.039827314	1.080064275	0.054773213	0.70094624
Q8N9R8	SCAI	Protein SCAI	0.996070085	0.98006003	-0.023377138	0.7073858
P15104	GLUL	Glutamine synthetase	0.964303509	0.987889526	0.034862419	0.71272162
Q9UHG2	PCSK1N	ProSAAS	1.098536034	1.074402091	-0.032048176	0.76114798
Q14894	CRYM	Ketimine reductase mu-crystallin	1.032169198	1.021445088	-0.015067834	0.76868143
P05771	PRKCB	Protein kinase C beta type	0.809437161	0.795711752	-0.024673178	0.820276
Q9H2X9	SLC12A5	Solute carrier family 12 member 5	1.002439378	1.017961611	0.022168164	0.85765623
P14854	COX6B1	Cytochrome c oxidase subunit 6B1	1.231708994	1.035887597	-0.249793976	0.20103366
Q9H2M9	RAB3GAP2	Rab3 GTPase-activating protein non-catalytic subunit	0.952531499	0.852728757	-0.159679893	0.36180439
P48169	GABRA4	Gamma-aminobutyric acid receptor subunit alpha-4	0.921257111	0.976828168	0.084500952	0.3952279
Q9UHG3	PCYOX1	Prenylcysteine oxidase 1	1.040791749	1.103461432	0.084354777	0.41332178
P17858	PFKL	ATP-dependent 6-phosphofructokinase, liver type	0.958664012	1.006947037	0.070890623	0.47487943
Q7L0J3	SV2A	Synaptic vesicle glycoprotein 2A	1.02958064	1.077535651	0.065678772	0.47687769
P51674	GPM6A	Neuronal membrane glycoprotein M6-a	1.030152758	1.13930434	0.145294897	0.49604037
P07203	GPX1	Glutathione peroxidase 1	0.947108894	0.912777342	-0.05326733	0.50604709
P31948	STIP1	Stress-induced-phosphoprotein 1	0.996412493	1.044524513	0.068031335	0.51974713

© 2020. Muraoka S et al. Published online at http://www.aginganddisease.org/EN/10.14336/AD.2020.1007

Q9HCJ6	VAT1L	Synaptic vesicle membrane protein VAT-1 homolog-like	0.911806266	0.981699392	0.106553999	0.53802028
P20936	RASA1	Ras GTPase-activating protein 1	0.890787208	0.847194246	-0.072388049	0.54634391
P13591	NCAM1	Neural cell adhesion molecule 1	0.993409613	0.957820113	-0.052633977	0.57148215
Q01082	SPTBN1	Spectrin beta chain, non-erythrocytic 1	0.93079878	0.954454119	0.036206529	0.59819432
Q9BT78	COPS4	COP9 signalosome complex subunit 4	0.93544429	0.977835631	0.06394024	0.60184186
P37235	HPCAL1	Hippocalcin-like protein 1	1.00026747	0.979248618	-0.030638735	0.60877535
P49418	AMPH	Amphiphysin	1.024006302	1.002636635	-0.03042574	0.61869004
Q9Y4L1	HYOU1	Hypoxia up-regulated protein 1	1.112171623	1.164693189	0.066570529	0.62072056
P30085	CMPK1	UMP-CMP kinase	0.938234674	0.909608532	-0.044703032	0.62272504
Q8N126	CADM3	Cell adhesion molecule 3	1.168014695	1.225452693	0.069256367	0.65336934
Q3ZCW2	LGALSL	Galectin-related protein	0.852977963	0.830143319	-0.039148038	0.65886681
Q9UBS5	GABBR1	Gamma-aminobutyric acid type B receptor subunit 1	0.80401649	0.754596736	-0.091519233	0.69627777
Q9H313	TTYH1	Protein tweety homolog 1	1.030797588	1.043525108	0.017704247	0.71956656
P61981	YWHAG	14-3-3 protein gamma	0.834126926	0.865798611	0.053764557	0.73332067
P11142	HSPA8	Heat shock cognate 71 kDa protein	0.990712162	1.009929979	0.027717402	0.74204
P61457	PCBD1	Pterin-4-alpha-carbinolamine dehydratase	0.808948434	0.770082293	-0.071035118	0.75505012
O14880	MGST3	Microsomal glutathione S-transferase 3	1.001311781	0.968050468	-0.048737093	0.75734069
P02792	FTL	Ferritin light chain	0.920053581	0.940593443	0.031853394	0.75839909
P60201	PLP1	Myelin proteolipid protein	1.14096735	1.219244393	0.09572983	0.76312129
Q14697	GANAB	Neutral alpha-glucosidase AB	1.021144731	1.058810327	0.052256812	0.76514817
P23468	PTPRD	Receptor-type tyrosine-protein phosphatase delta	0.949359812	0.938381844	-0.016779879	0.79489169
Q15365	PCBP1	Poly(rC)-binding protein 1	0.967396182	0.990186639	0.033593639	0.79940571
P10301	RRAS	Ras-related protein R-Ras	1.008558946	0.992779698	-0.022749889	0.83478561
Q15149	PLEC	Plectin	0.988976192	0.997648167	0.01259533	0.83596063
O15075	DCLK1	Serine/threonine-protein kinase DCLK1	0.982567392	0.972142077	-0.015389186	0.83780497
Q99460	PSMD1	26S proteasome non-ATPase regulatory subunit 1	1.011509099	0.989317931	-0.032003166	0.84707522
P04406	GAPDH	Glyceraldehyde-3-phosphate dehydrogenase	0.865745965	0.874414747	0.014373974	0.85318067
Q9UM22	EPDR1	Mammalian ependymin-related protein 1	1.018741997	1.005573751	-0.01876983	0.85618313
P61586	RHOA	Transforming protein RhoA	0.984702307	0.992450981	0.011308209	0.85984902
Q9H299	SH3BGRL3	SH3 domain-binding glutamic acid-rich-like protein 3	0.89880585	0.884415853	-0.023284629	0.87851704
P07355	ANXA2	Annexin A2	0.971459233	0.957196685	-0.021338055	0.8800228
Q14156	EFR3A	Protein EFR3 homolog A	0.936935662	0.958195505	0.032370061	0.91289581
Q9Y2J2	EPB41L3	Band 4.1-like protein 3	0.929865326	0.943622901	0.021188649	0.91381151
Q14642	INPP5A	Type I inositol 1,4,5-trisphosphate 5-phosphatase	0.810181066	0.798436114	-0.021067394	0.91867531
Q8IZP0	ABI1	Abl interactor 1	0.951560688	0.958001961	0.009732938	0.93046303
O60262	GNG7	Guanine nucleotide-binding protein G(I)/G(S)/G(O) subunit gamma-7	0.947616073	0.94161153	-0.009170684	0.93126266
Q9UJU6	DBNL	Drebrin-like protein	0.945229485	1.029726086	0.123524084	0.44060201
P52209	PGD	6-phosphogluconate dehydrogenase, decarboxylating	1.292823708	1.140773512	-0.18051317	0.47506953
Q9BTU6	PI4K2A	Phosphatidylinositol 4-kinase type 2-alpha	1.065005342	0.994060144	-0.09945562	0.4831287

© 2020. Muraoka S et al. Published online at http://www.aginganddisease.org/EN/10.14336/AD.2020.1007

SUPPLEMENTARY DATA

P36405	ARL3	ADP-ribosylation factor-like protein 3	0.963812012	0.913266921	-0.077715204	0.49119433
P61026	RAB10	Ras-related protein Rab-10	0.946309031	1.015582999	0.101924851	0.50002155
P60953	CDC42	Cell division control protein 42 homolog	0.917981303	0.96466487	0.071563059	0.51760713
O94919	ENDOD1	Endonuclease domain-containing 1 protein	1.044258623	1.018969247	-0.035368546	0.54433035
Q9HAV0	GNB4	Guanine nucleotide-binding protein subunit beta-4	1.073580933	1.139255067	0.085659834	0.57190596
O75899	GABBR2	Gamma-aminobutyric acid type B receptor subunit 2	1.148058154	1.102220692	-0.058782606	0.59045737
P19086	GNAZ	Guanine nucleotide-binding protein G(z) subunit alpha	1.074511939	1.150658583	0.098778315	0.59793368
P07339	CTSD	Cathepsin D	1.046750254	1.12846248	0.108441183	0.60239163
Q7L576	CYFIP1	Cytoplasmic FMR1-interacting protein 1	0.998871506	1.016754055	0.025599736	0.62240015
Q8TDJ6	DMXL2	DmX-like protein 2	0.960961268	0.931175442	-0.045425274	0.68558407
Q9NP72	RAB18	Ras-related protein Rab-18	0.923265166	0.893403304	-0.047433465	0.68615736
Q9Y617	PSAT1	Phosphoserine aminotransferase	0.940009991	0.977354333	0.056205605	0.68901764
P68871	HBB	Hemoglobin subunit beta	0.9588764	0.993274866	0.050848142	0.7241499
P17066	HSPA6	Heat shock 70 kDa protein 6	0.972789266	1.000462038	0.04046721	0.73199415
P59998	ARPC4	Actin-related protein 2/3 complex subunit 4	0.952682165	0.962224364	0.014378349	0.73294665
P42858	HTT	Huntingtin	1.002084531	1.020473286	0.026234203	0.77643219
O43236	SEP4	Septin-4	0.769291041	0.744168396	-0.047900384	0.84175599
Q96RR4	CAMKK2	Calcium/calmodulin-dependent protein kinase kinase 2	1.129474512	1.104787425	-0.031882911	0.84613301
Q9UPN3	MACF1	Microtubule-actin cross-linking factor 1, isoforms 1/2/3/5	0.958740373	0.967511217	0.013138202	0.87336973
Q99878	HIST1H2AJ	Histone H2A type 1-J	1.332816669	1.308013123	-0.027101335	0.9027415
Q9H115	NAPB	Beta-soluble NSF attachment protein	0.931806865	0.939377159	0.011673555	0.90684385
O75335	PPFIA4	Liprin-alpha-4	0.940347345	0.948951878	0.013141172	0.91005623
Q15811	ITSN1	Intersectin-1	1.05431105	1.046784433	-0.010336188	0.91903675
Q13813	SPTAN1	Spectrin alpha chain, non-erythrocytic 1	0.962086266	0.965991974	0.005844943	0.92401978
Q9H8M5	CNNM2	Metal transporter CNNM2	0.999401799	0.990098791	-0.013492331	0.92841731
Q9P2U7	SLC17A7	Vesicular glutamate transporter 1	1.035672868	1.038831513	0.004393305	0.95221595
P68036	UBE2L3	Ubiquitin-conjugating enzyme E2 L3	1.058887314	1.063960304	0.006895258	0.95665174
P62987	UBA52	Ubiquitin-60S ribosomal protein L40	0.858148109	0.861358893	0.005387808	0.97853762
P00558	PGK1	Phosphoglycerate kinase 1	1.033152528	1.035360774	0.003080306	0.97885264
P43003	SLC1A3	Excitatory amino acid transporter 1	1.028235952	1.024516641	-0.005227944	0.98220054
P23381	WARS	Tryptophan--tRNA ligase, cytoplasmic	1.01901396	1.02044034	0.002018023	0.98859842
P78559	MAP1A	Microtubule-associated protein 1A	1.112493303	1.040165261	-0.09698389	0.33024439
P30041	PRDX6	Peroxiredoxin-6	0.961720348	1.006258018	0.06531093	0.38750031
P78371	CCT2	T-complex protein 1 subunit beta	1.030798714	1.058393519	0.038113491	0.45070568
P01303	NPY	Pro-neuropeptide Y	0.998079186	0.935574818	-0.093301251	0.45154191
Q16653	MOG	Myelin-oligodendrocyte glycoprotein	1.084757152	1.383163233	0.350599327	0.46081655
Q01484	ANK2	Ankyrin-2	0.998836428	0.970429158	-0.04162554	0.51835986
P40123	CAP2	Adenylyl cyclase-associated protein 2	0.978821128	1.033044823	0.077785705	0.53413401
P31939	ATIC	Bifunctional purine biosynthesis protein PURH	0.980328447	1.017623104	0.053866237	0.55312712

© 2020. Muraoka S et al. Published online at http://www.aginganddisease.org/EN/10.14336/AD.2020.1007

P62937	PPIA	Peptidyl-prolyl cis-trans isomerase A	0.905235278	0.946922264	0.064953186	0.59535736
P00492	HPRT1	Hypoxanthine-guanine phosphoribosyltransferase	0.906136569	0.86449509	-0.067870733	0.60436587
O43157	PLXNB1	Plexin-B1	1.221430029	1.270607007	0.056946661	0.63872551
P22694	PRKACB	cAMP-dependent protein kinase catalytic subunit beta	0.967288783	0.956707121	-0.015869331	0.6749739
P02763	ORM1	Alpha-1-acid glycoprotein 1	0.923236093	0.971879306	0.074077536	0.67670267
P43034	PAFAH1B1	Platelet-activating factor acetylhydrolase IB subunit alpha	1.016259638	1.045143497	0.040432003	0.67966766
P31323	PRKAR2B	cAMP-dependent protein kinase type II-beta regulatory subunit	0.989808188	1.005799628	0.023122043	0.71133246
Q13153	PAK1	Serine/threonine-protein kinase PAK 1	1.13895777	1.220166466	0.09936373	0.74609023
P04792	HSPB1	Heat shock protein beta-1	0.984682974	1.006680899	0.031875228	0.75624336
P50993	ATP1A2	Sodium/potassium-transporting ATPase subunit alpha-2	0.963949532	0.955810883	-0.01223242	0.79103144
Q16643	DBN1	Drebrin	0.894020478	0.8811658	-0.020894376	0.80853493
Q9BQI5	SGIP1	SH3-containing GRB2-like protein 3-interacting protein 1	1.020898475	1.00409676	-0.0239411	0.81197823
P61764	STXBP1	Syntaxin-binding protein 1	0.990141814	1.004110586	0.02021109	0.81763031
Q15084	PDIA6	Protein disulfide-isomerase A6	1.021879509	1.040741749	0.026387025	0.82038545
P43007	SLC1A4	Neutral amino acid transporter A	1.041828939	1.065020423	0.031762679	0.84340184
Q9NZQ3	NCKIPSD	NCK-interacting protein with SH3 domain	0.901403555	0.912809378	0.018140474	0.8763386
Q9P035	HACD3	Very-long-chain (3R)-3-hydroxyacyl-CoA dehydratase 3	0.916254637	0.929502162	0.020709627	0.88744817
Q15286	RAB35	Ras-related protein Rab-35	0.872231359	0.880689147	0.013922028	0.88831387
P11166	SLC2A1	Solute carrier family 2, facilitated glucose transporter member 1	0.907652445	0.936919229	0.045784708	0.88865022
P21281	ATP6V1B2	V-type proton ATPase subunit B, brain isoform	0.907360379	0.912953495	0.008865708	0.90839836
P48066	SLC6A11	Sodium- and chloride-dependent GABA transporter 3	0.943605725	0.950418478	0.010378714	0.93464992
P08238	HSP90AB1	Heat shock protein HSP 90-beta	1.040270779	1.04234232	0.002870051	0.96974421
P18507	GABRG2	Gamma-aminobutyric acid receptor subunit gamma-2	1.060311656	1.060243043	-9.336E-05	0.99960802
O75436	VPS26A	Vacuolar protein sorting-associated protein 26A	0.966015407	1.044679789	0.112942696	0.52219454
O00139	KIF2A	Kinesin-like protein KIF2A	1.198737893	1.077465001	-0.153875237	0.52599284
O95670	ATP6V1G2	V-type proton ATPase subunit G 2	0.987562097	1.030998776	0.062099249	0.55858005
P42263	GRIA3	Glutamate receptor 3	1.032108278	1.084074563	0.070869657	0.61330481
Q16537	PPP2R5E	Serine/threonine-protein phosphatase 2A 56 kDa regulatory subunit epsilon isoform	0.904582967	0.934259663	0.046570749	0.62749967
Q92871	PMM1	Phosphomannomutase 1	0.934420912	0.974220264	0.06017543	0.6342303
P14415	ATP1B2	Sodium/potassium-transporting ATPase subunit beta-2	1.069908231	1.198242609	0.163432983	0.6443638
P26885	FKBP2	Peptidyl-prolyl cis-trans isomerase FKBP2	0.880562899	0.92074666	0.0643782	0.65505049
Q8TCU6	PREX1	Phosphatidylinositol 3,4,5-trisphosphate-dependent Rac exchanger 1 protein	1.12825646	1.190174261	0.077077783	0.6689053
Q9UKU0	ACSL6	Long-chain-fatty-acid--CoA ligase 6	1.06710559	1.03590129	-0.042816402	0.70032345
Q32P51	HNRNPA1L2	Heterogeneous nuclear ribonucleoprotein A1-like 2	1.006811938	0.978982794	-0.040438818	0.74203669
P13716	ALAD	Delta-aminolevulinic acid dehydratase	0.95092069	0.972658922	0.03260897	0.74258188
Q9UH03	SEP3	Neuronal-specific septin-3	0.885545487	0.899699021	0.022876038	0.78555027
P21397	MAOA	Amine oxidase [flavin-containing] A	1.124450195	1.104924559	-0.025271892	0.7863816
P42658	DPP6	Dipeptidyl aminopeptidase-like protein 6	1.021219858	1.032079565	0.015260699	0.81395594

© 2020. Muraoka S et al. Published online at http://www.aginganddisease.org/EN/10.14336/AD.2020.1007

P62333	PSMC6	26S protease regulatory subunit 10B	1.004232927	0.985715032	-0.026851402	0.81819692
Q01469	FABP5	Fatty acid-binding protein, epidermal	1.02916931	1.018822732	-0.014577288	0.82348064
P02686	MBP	Myelin basic protein	1.00746326	0.988030025	-0.028100439	0.83540151
Q10567	AP1B1	AP-1 complex subunit beta-1	0.94843234	0.933589959	-0.022755813	0.83980226
P62258	YWHAE	14-3-3 protein epsilon	1.045908336	1.062093709	0.022154643	0.85597674
Q15121	PEA15	Astrocytic phosphoprotein PEA-15	1.187198792	1.175437422	-0.014363794	0.85980432
P50995	ANXA11	Annexin A11	1.070417317	1.064715669	-0.007705149	0.88984449
Q86YS7	C2CD5	C2 domain-containing protein 5	1.126330286	1.103521449	-0.029515276	0.90782931
P23763	VAMP1	Vesicle-associated membrane protein 1	0.895080115	0.885778585	-0.015070701	0.91974689
P04083	ANXA1	Annexin A1	0.982271023	0.98860505	0.009273135	0.92002233
Q15019	SEP2	Septin-2	0.89840487	0.902633466	0.006774521	0.92558524
P04179	SOD2	Superoxide dismutase [Mn], mitochondrial	0.94728056	0.953429104	0.009333887	0.93250295
P63092	GNAS	Guanine nucleotide-binding protein G(s) subunit alpha isoforms short	0.947043347	0.943848596	-0.004875007	0.935863
P68402	PAFAH1B2	Platelet-activating factor acetylhydrolase IB subunit beta	0.573672154	0.587274936	0.033809575	0.93838367
P05129	PRKCG	Protein kinase C gamma type	0.903883521	0.908532331	0.007400983	0.95247314
Q9Y2J8	PADI2	Protein-arginine deiminase type-2	1.089100709	1.091024505	0.00254614	0.96810733
P38646	HSPA9	Stress-70 protein, mitochondrial	1.114612492	1.11304702	-0.002027688	0.97465624
P16949	STMN1	Stathmin	1.016227582	1.017036158	0.001147444	0.98988816
P23526	AHCY	Adenosylhomocysteinase	1.029995672	1.029019306	-0.001368225	0.99185568
Q96CX2	KCTD12	BTB/POZ domain-containing protein KCTD12	1.027048605	1.026627337	-0.000591875	0.99715777
Q16623	STX1A	Syntaxin-1A	0.95789853	0.957893899	-6.97454E-06	0.99992945
Q9Y2I8	WDR37	WD repeat-containing protein 37	1.027024128	1.079774255	0.072259649	0.53559528
P67775	PPP2CA	Serine/threonine-protein phosphatase 2A catalytic subunit alpha isoform	0.988143642	1.027453464	0.056280372	0.5372854
P08195	SLC3A2	4F2 cell-surface antigen heavy chain	0.932010361	0.979049128	0.071035262	0.58132989
P11177	PDHB	Pyruvate dehydrogenase E1 component subunit beta, mitochondrial	0.947681582	0.975452151	0.041668706	0.64025663
Q14832	GRM3	Metabotropic glutamate receptor 3	1.190853754	1.237131235	0.0550023	0.66119543
P46821	MAP1B	Microtubule-associated protein 1B	0.941389319	0.926318151	-0.023283702	0.75752301
P11137	MAP2	Microtubule-associated protein 2	1.066691626	1.089471035	0.030484679	0.76815523
P35580	MYH10	Myosin-10	1.075813967	1.095809936	0.026568966	0.77260038
P78357	CNTNAP1	Contactin-associated protein 1	0.984296281	0.972791052	-0.016962684	0.77397693
Q08209	PPP3CA	Serine/threonine-protein phosphatase 2B catalytic subunit alpha isoform	1.096513199	1.130703638	0.044297665	0.77610032
Q86VP6	CAND1	Cullin-associated NEDD8-dissociated protein 1	1.041619528	1.018930844	-0.031772263	0.80527355
P00441	SOD1	Superoxide dismutase [Cu-Zn]	0.977014057	0.964888552	-0.018017004	0.82656995
Q86TV6	TTC7B	Tetratricopeptide repeat protein 7B	0.857917142	0.843061882	-0.025199787	0.84882104
P62760	VSNL1	Visinin-like protein 1	0.959865601	0.953847607	-0.009073627	0.8930735
P61225	RAP2B	Ras-related protein Rap-2b	1.178126863	1.151762335	-0.032651851	0.9028149
Q9UQ16	DNM3	Dynamin-3	1.076891711	1.086119256	0.012309336	0.90781484
Q9HDC9	APMAP	Adipocyte plasma membrane-associated protein	1.035816609	1.040325767	0.006266767	0.95770426
Q13636	RAB31	Ras-related protein Rab-31	0.860098424	0.904103037	0.071985438	0.48468362

© 2020. Muraoka S et al. Published online at http://www.aginganddisease.org/EN/10.14336/AD.2020.1007

Q9UN36	NDRG2	Protein NDRG2	1.029203441	1.13567917	0.142027144	0.52831519
P47870	GABRB2	Gamma-aminobutyric acid receptor subunit beta-2	1.01207298	0.980894119	-0.045144005	0.56020987
P02730	SLC4A1	Band 3 anion transport protein	0.856358244	0.930426148	0.11967719	0.61205964
Q9Y6R1	SLC4A4	Electrogenic sodium bicarbonate cotransporter 1	1.064492823	1.146650603	0.10725963	0.64472437
P08247	SYP	Synaptophysin	0.887832049	0.854574139	-0.05508113	0.65864621
O15254	ACOX3	Peroxisomal acyl-coenzyme A oxidase 3	1.000599934	0.958502959	-0.062010471	0.66490175
Q15819	UBE2V2	Ubiquitin-conjugating enzyme E2 variant 2	0.975344246	0.947673604	-0.041521252	0.70780844
Q02978	SLC25A11	Mitochondrial 2-oxoglutarate/malate carrier protein	0.963906197	1.03202288	0.098510293	0.71527695
P51659	HSD17B4	Peroxisomal multifunctional enzyme type 2	1.058454395	1.075463874	0.022999954	0.72242897
P28070	PSMB4	Proteasome subunit beta type-4	1.063424648	1.032268481	-0.042899563	0.75709851
O95782	AP2A1	AP-2 complex subunit alpha-1	0.92836649	0.914387164	-0.021889297	0.79491297
O43813	LANCL1	LanC-like protein 1	0.943812406	0.951796571	0.012153122	0.81454551
P43304	GPD2	Glycerol-3-phosphate dehydrogenase, mitochondrial	1.035383921	1.018219697	-0.02411694	0.84977161
P50990	CCT8	T-complex protein 1 subunit theta	0.992624702	1.000709114	0.01170241	0.85054918
P35222	CTNNB1	Catenin beta-1	1.091721863	1.082127359	-0.012735044	0.85416796
Q9P2D8	UNC79	Protein unc-79 homolog	0.998344361	1.016003001	0.025295225	0.86611321
P04216	THY1	Thy-1 membrane glycoprotein	1.006659544	0.993087095	-0.019583687	0.87289031
Q92561	PHYHIP	Phytanoyl-CoA hydroxylase-interacting protein	1.080545586	1.104677305	0.031865057	0.87337719
Q9BVA1	TUBB2B	Tubulin beta-2B chain	1.026531076	1.011804398	-0.020846887	0.87936217
P46459	NSF	Vesicle-fusing ATPase	0.945293777	0.939662383	-0.008620262	0.88147444
Q96CW1	AP2M1	AP-2 complex subunit mu	0.98146203	0.987904766	0.009439518	0.88298737
P06576	ATP5B	ATP synthase subunit beta, mitochondrial	0.91695938	0.904300059	-0.020056269	0.89829292
Q16181	SEP7	Septin-7	0.909586776	0.918058423	0.013374687	0.90205094
Q9BR01	SULT4A1	Sulfotransferase 4A1	1.102940515	1.093372696	-0.01256973	0.92549859
Q93050	ATP6V0A1	V-type proton ATPase 116 kDa subunit a isoform 1	0.99630315	0.999878115	0.005167456	0.93055833
Q92686	NRGN	Neurogranin	0.920984454	0.931787822	0.016824671	0.93390678
O75396	SEC22B	Vesicle-trafficking protein SEC22b	0.907143586	0.901979947	-0.008235563	0.95655172
Q13509	TUBB3	Tubulin beta-3 chain	0.989483476	0.994959836	0.007962675	0.96048068
P0DMV9	HSPA1B	Heat shock 70 kDa protein 1B	0.967748099	0.970947646	0.004761939	0.96448313
P68371	TUBB4B	Tubulin beta-4B chain	0.78403983	0.782465481	-0.002899839	0.97884856
P80404	ABAT	4-aminobutyrate aminotransferase, mitochondrial	0.905620367	0.908633002	0.0047913	0.98132064
P06756	ITGAV	Integrin alpha-V	0.942189607	0.942567981	0.000579256	0.99566391
Q9H0E2	TOLLIP	Toll-interacting protein	0.990113296	0.989982271	-0.000190928	0.99873511

[a] The *p* .value was calculated by student's t. test.

© 2020. Muraoka S et al. Published online at http://www.aginganddisease.org/EN/10.14336/AD.2020.1007

Supplementary Table 3. Protein list of these modules which were significantly positive correlated with t-tau in WPCNA.

Module	Uniprot ID	Gene Name	Correlation coefficient with t-tau	*p*-value
M7	P10599	TXN	0.513	0.029421737
M7	P31150	GDI1	0.140	0.579155302
M7	P49368	CCT3	0.527	0.024632834
M7	P48147	PREP	0.267	0.283658808
M7	Q96CS3	FAF2	0.453	0.059242581
M7	O00154	ACOT7	-0.123	0.627977179
M7	O15145	ARPC3	0.206	0.413298627
M7	P14867	GABRA1	-0.215	0.392357649
M7	P47755	CAPZA2	0.387	0.113077719
M7	P32119	PRDX2	0.117	0.645154981
M7	P17858	PFKL	0.523	0.025809951
M7	Q9BT78	COPS4	0.201	0.423520693
M7	P10301	RRAS	0.364	0.137758387
M7	Q9UM22	EPDR1	-0.546	0.019082681
M7	Q9UJU6	DBNL	0.450	0.060813274
M7	P60953	CDC42	0.488	0.039893026
M7	O75899	GABBR2	-0.601	0.008366007
M7	P02763	ORM1	0.745	0.000390844
M7	Q9UH03	SEP3	0.416	0.085842439
M8	Q96P70	IPO9	0.651	0.003453692
M8	P08237	PFKM	0.515	0.028557577
M8	Q9GZN7	ROGDI	0.531	0.023209365
M8	Q92597	NDRG1	0.323	0.191425244
M8	O75955	FLOT1	0.618	0.006319354
M8	Q07960	ARHGAP1	0.604	0.007930549
M8	P21266	GSTM3	0.541	0.020427979
M8	Q16348	SLC15A2	-0.459	0.055174269
M8	Q9Y4L1	HYOU1	0.256	0.304717015
M8	Q9UPN3	MACF1	0.450	0.060847528
M8	P68036	UBE2L3	0.422	0.081043724
M8	Q92871	PMM1	0.635	0.004657478
M8	Q01469	FABP5	-0.245	0.327488491
M8	Q96CX2	KCTD12	0.600	0.008425607
M8	Q9Y2I8	WDR37	0.414	0.088023299
M8	Q9Y6R1	SLC4A4	0.377	0.122567177
M8	O15254	ACOX3	-0.577	0.01214402
M9	P05023	ATP1A1	0.281	0.25801866

© 2020. Muraoka S et al. Published online at http://www.aginganddisease.org/EN/10.14336/AD.2020.1007

M9	Q01518	CAP1	0.764	0.000223132
M9	P61266	STX1B	0.321	0.193597036
M9	P29401	TKT	0.565	0.01457533
M9	Q9BY11	PACSIN1	0.420	0.083062741
M9	O14807	MRAS	0.475	0.04653766
M9	Q13098	GPS1	0.574	0.012660324
M9	P55072	VCP	0.609	0.007341998
M9	Q16658	FSCN1	0.405	0.095215109
M9	Q92777	SYN2	0.298	0.229427262
M9	P00338	LDHA	0.347	0.158116541
M9	P62879	GNB2	0.504	0.03296379
M9	Q14195	DPYSL3	0.290	0.243031543
M9	P32418	SLC8A1	0.609	0.007303928
M9	P62826	RAN	0.433	0.0725384
M9	Q01814	ATP2B2	0.577	0.012210381
M9	Q06124	PTPN11	-0.164	0.515156711
M9	Q9NRW1	RAB6B	0.703	0.001145438
M9	P08559	PDHA1	-0.681	0.001858551
M9	Q92599	SEP8	0.533	0.022874309
M9	P07203	GPX1	0.215	0.390635238
M9	Q01082	SPTBN1	0.428	0.076161132
M9	P61981	YWHAG	0.552	0.017609773
M9	P23468	PTPRD	0.660	0.002902167
M9	Q8IZP0	ABI1	0.609	0.0073372
M9	P42858	HTT	0.496	0.036300331
M9	Q13813	SPTAN1	0.446	0.063471629
M9	P62987	UBA52	0.643	0.003991753
M9	O43157	PLXNB1	0.183	0.466898744
M9	P31323	PRKAR2B	-0.343	0.163705116
M9	Q15286	RAB35	0.226	0.366586188
M9	P48066	SLC6A11	-0.768	0.000195714
M9	P16949	STMN1	0.639	0.00428614
M9	Q16623	STX1A	0.385	0.11478435
M9	P46821	MAP1B	0.605	0.007825444
M9	Q9HDC9	APMAP	-0.386	0.113646658
M9	P08247	SYP	0.443	0.065458088
M9	Q15819	UBE2V2	0.711	0.000950248
M9	Q9BVA1	TUBB2B	-0.467	0.050875058
M9	P46459	NSF	0.544	0.019513023
M9	Q9BR01	SULT4A1	0.415	0.086856991
M9	Q93050	ATP6V0A1	-0.754	0.00030348
M9	Q92686	NRGN	0.717	0.000809369

© 2020. Muraoka S et al. Published online at http://www.aginganddisease.org/EN/10.14336/AD.2020.1007

SUPPLEMENTARY DATA

Supplementary Table 4. Gene ontology terms enrichment for M7, M8, M9, M10 and M11 modules in WPCNA.

Module	Rank	Enrichment p.value	Term ID	Term Ontology	Term Name	Term Definition
M7	1	0.001297017	GO:0090317	BP	negative regulation of intracellular protein transport	Any process that decreases the frequency, rate or extent of the directed movement of proteins within cells.
M7	2	0.003136841	GO:0051224	BP	negative regulation of protein transport	Any process that stops, prevents, or reduces the frequency, rate or extent of the directed movement of a protein into, out of or within a cell, or between cells, by means of some agent such as a transporter or pore.
M7	3	0.004376024	GO:1904950	BP	negative regulation of establishment of protein localization	NA
M7	4	0.004455869	GO:0110053	BP	regulation of actin filament organization	Any process that modulates the frequency, rate or extent of actin filament organization.
M7	5	0.00757723	GO:0032956	BP	regulation of actin cytoskeleton organization	Any process that modulates the frequency, rate or extent of the formation, arrangement of constituent parts, or disassembly of cytoskeletal structures comprising actin filaments and their associated proteins.
M7	6	0.0120714	GO:0032147	BP	activation of protein kinase activity	Any process that initiates the activity of an inactive protein kinase.
M7	7	0.012128936	GO:0032387	BP	negative regulation of intracellular transport	Any process that stops, prevents, or reduces the frequency, rate or extent of the directed movement of substances within cells.
M7	8	0.013638845	GO:0032970	BP	regulation of actin filament-based process	Any process that modulates the frequency, rate or extent of any cellular process that depends upon or alters the actin cytoskeleton.
M7	9	0.017710713	GO:0008064	BP	regulation of actin polymerization or depolymerization	Any process that modulates the frequency, rate or extent of the assembly or disassembly of actin filaments by the addition or removal of actin monomers from a filament.
M7	10	0.017710713	GO:0030832	BP	regulation of actin filament length	Any process that controls the length of actin filaments in a cell.
M8	1	0.001031546	GO:0005504	MF	fatty acid binding	Interacting selectively and non-covalently with fatty acids, aliphatic monocarboxylic acids liberated from naturally occurring fats and oils by hydrolysis.

© 2020. Muraoka S et al. Published online at http://www.aginganddisease.org/EN/10.14336/AD.2020.1007

M8	2	0.00972337	GO:0007422	BP	peripheral nervous system development	The process whose specific outcome is the progression of the peripheral nervous system over time, from its formation to the mature structure. The peripheral nervous system is one of the two major divisions of the nervous system. Nerves in the PNS connect the central nervous system (CNS) with sensory organs, other organs, muscles, blood vessels and glands.
M8	3	0.014301096	GO:0044242	BP	cellular lipid catabolic process	The chemical reactions and pathways resulting in the breakdown of lipids, as carried out by individual cells.
M8	4	0.014301096	GO:0031406	MF	carboxylic acid binding	Interacting selectively and non-covalently with a carboxylic acid, any organic acid containing one or more carboxyl (COOH) groups or anions (COO-).
M8	5	0.025667733	GO:0015293	MF	symporter activity	Enables the active transport of a solute across a membrane by a mechanism whereby two or more species are transported together in the same direction in a tightly coupled process not directly linked to a form of energy other than chemiosmotic energy.
M8	6	0.025667733	GO:0043177	MF	organic acid binding	Interacting selectively and non-covalently with an organic acid, any acidic compound containing carbon in covalent linkage.
M8	7	0.032361046	GO:1901888	BP	regulation of cell junction assembly	NA
M8	8	0.03307393	GO:0001963	BP	synaptic transmission, dopaminergic	The vesicular release of dopamine. from a presynapse, across a chemical synapse, the subsequent activation of dopamine receptors at the postsynapse of a target cell (neuron, muscle, or secretory cell) and the effects of this activation on the postsynaptic membrane potential and ionic composition of the postsynaptic cytosol. This process encompasses both spontaneous and evoked release of neurotransmitter and all parts of synaptic vesicle exocytosis. Evoked transmission starts with the arrival of an action potential at the presynapse.
M8	9	0.03307393	GO:0006013	BP	mannose metabolic process	The chemical reactions and pathways involving mannose, the aldohexose manno-hexose, the C-2 epimer of glucose. The D-(+)-form is widely distributed in mannans and hemicelluloses and is of major importance in the core oligosaccharide of N-linked oligosaccharides of glycoproteins.

© 2020. Muraoka S et al. Published online at http://www.aginganddisease.org/EN/10.14336/AD.2020.1007

M8	10	0.03307393	GO:0006857	BP	oligopeptide transport	The directed movement of oligopeptides into, out of or within a cell, or between cells, by means of some agent such as a transporter or pore. Oligopeptides are molecules that contain a small number (2 to 20) of amino-acid residues connected by peptide linkages.
M9	1	0.000274847	GO:0019904	MF	protein domain specific binding	Interacting selectively and non-covalently with a specific domain of a protein.
M9	2	0.000281676	GO:0045202	CC	synapse	The junction between an axon of one neuron and a dendrite of another neuron, a muscle fiber or a glial cell. As the axon approaches the synapse it enlarges into a specialized structure, the presynaptic terminal bouton, which contains mitochondria and synaptic vesicles. At the tip of the terminal bouton is the presynaptic membrane; facing it, and separated from it by a minute cleft (the synaptic cleft) is a specialized area of membrane on the receiving cell, known as the postsynaptic membrane. In response to the arrival of nerve impulses, the presynaptic terminal bouton secretes molecules of neurotransmitters into the synaptic cleft. These diffuse across the cleft and transmit the signal to the postsynaptic membrane.
M9	3	0.000368293	GO:0030054	CC	cell junction	A cellular component that forms a specialized region of connection between two or more cells, or between a cell and the extracellular matrix, or between two membrane-bound components of a cell, such as flagella.
M9	4	0.00050049	GO:0006996	BP	organelle organization	A process that is carried out at the cellular level which results in the assembly, arrangement of constituent parts, or disassembly of an organelle within a cell. An organelle is an organized structure of distinctive morphology and function. Includes the nucleus, mitochondria, plastids, vacuoles, vesicles, ribosomes and the cytoskeleton. Excludes the plasma membrane.
M9	5	0.000548468	GO:0006301	BP	postreplication repair	The conversion of DNA-damage induced single-stranded gaps into large molecular weight DNA after replication. Includes pathways that remove replication-blocking lesions in conjunction with DNA replication.
M9	6	0.001491373	GO:0032984	BP	protein-containing complex disassembly	The disaggregation of a protein-containing macromolecular complex into its constituent components.

© 2020. Muraoka S et al. Published online at http://www.aginganddisease.org/EN/10.14336/AD.2020.1007

M9	7	0.001733952	GO:0023052	BP	signaling	The entirety of a process in which information is transmitted within a biological system. This process begins with an active signal and ends when a cellular response has been triggered.
M9	8	0.002217193	GO:0007010	BP	cytoskeleton organization	A process that is carried out at the cellular level which results in the assembly, arrangement of constituent parts, or disassembly of cytoskeletal structures.
M9	9	0.002335705	GO:0006281	BP	DNA repair	The process of restoring DNA after damage. Genomes are subject to damage by chemical and physical agents in the environment (e.g. UV and ionizing radiations, chemical mutagens, fungal and bacterial toxins, etc.) and by free radicals or alkylating agents endogenously generated in metabolism. DNA is also damaged because of errors during its replication. A variety of different DNA repair pathways have been reported that include direct reversal, base excision repair, nucleotide excision repair, photoreactivation, bypass, double-strand break repair pathway, and mismatch repair pathway.
M9	10	0.002764995	GO:0014069	CC	postsynaptic density	An electron dense network of proteins within and adjacent to the postsynaptic membrane of an asymmetric, neuron-neuron synapse. Its major components include neurotransmitter receptors and the proteins that spatially and functionally organize them such as anchoring and scaffolding molecules, signaling enzymes and cytoskeletal components.
M10	1	0.0000316	GO:0019905	MF	syntaxin binding	Interacting selectively and non-covalently with a syntaxin, a SNAP receptor involved in the docking of synaptic vesicles at the presynaptic zone of a synapse.
M10	2	0.000139475	GO:0000149	MF	SNARE binding	Interacting selectively and non-covalently with a SNARE (soluble N-ethylmaleimide-sensitive factor attached protein receptor) protein.
M10	3	0.000560425	GO:0008021	CC	synaptic vesicle	A secretory organelle, typically 50 nm in diameter, of presynaptic nerve terminals; accumulates in high concentrations of neurotransmitters and secretes these into the synaptic cleft by fusion with the 'active zone' of the presynaptic plasma membrane.

© 2020. Muraoka S et al. Published online at http://www.aginganddisease.org/EN/10.14336/AD.2020.1007

M10	4	0.000662186	GO:0070382	CC	exocytic vesicle	A transport vesicle that mediates transport from an intracellular compartment to the plasma membrane, and fuses with the plasma membrane to release various cargo molecules, such as proteins or hormones, by exocytosis.
M10	5	0.001161138	GO:0017075	MF	syntaxin-1 binding	Interacting selectively and non-covalently with the SNAP receptor syntaxin-1.
M10	6	0.002376727	GO:0030133	CC	transport vesicle	Any of the vesicles of the constitutive secretory pathway, which carry cargo from the endoplasmic reticulum to the Golgi, between Golgi cisternae, from the Golgi to the ER (retrograde transport) or to destinations within or outside the cell.
M10	7	0.003408936	GO:0071156	BP	regulation of cell cycle arrest	Any process that modulates the rate, frequency, or extent of cell cycle arrest, the process in which the cell cycle is halted during one of the normal phases.
M10	8	0.003408936	GO:0070032	CC	synaptobrevin 2-SNAP-25-syntaxin-1a-complexin I complex	A SNARE complex that contains synaptobrevin 2 (VAMP2), SNAP-25, syntaxin 1a, and complexin I (or orthologs thereof).
M10	9	0.003408936	GO:0004843	MF	thiol-dependent ubiquitin-specific protease activity	Catalysis of the thiol-dependent hydrolysis of a peptide bond formed by the C-terminal glycine of ubiquitin and another protein.
M10	10	0.003408936	GO:0036459	MF	thiol-dependent ubiquitinyl hydrolase activity	Catalysis of the thiol-dependent hydrolysis of an ester, thioester, amide, peptide or isopeptide bond formed by the C-terminal glycine of ubiquitin.
M11	1	0.01084545	GO:0035094	BP	response to nicotine	Any process that results in a change in state or activity of a cell or an organism (in terms of movement, secretion, enzyme production, gene expression, etc.) as a result of a nicotine stimulus.
M11	2	0.01084545	GO:0072686	CC	mitotic spindle	A spindle that forms as part of mitosis. Mitotic and meiotic spindles contain distinctive complements of proteins associated with microtubules.
M11	3	0.01084545	GO:0005522	MF	profilin binding	Interacting selectively and non-covalently with profilin, an actin-binding protein that forms a complex with G-actin and prevents it from polymerizing to form F-actin.
M11	4	0.014277445	GO:0030055	CC	cell-substrate junction	A cell junction that forms a connection between a cell and the extracellular matrix.

© 2020. Muraoka S et al. Published online at http://www.aginganddisease.org/EN/10.14336/AD.2020.1007

M11	5	0.020846687	GO:0042475	BP	odontogenesis of dentin-containing tooth	The process whose specific outcome is the progression of a dentin-containing tooth over time, from its formation to the mature structure. A dentin-containing tooth is a hard, bony organ borne on the jaw or other bone of a vertebrate, and is composed mainly of dentin, a dense calcified substance, covered by a layer of enamel.
M11	6	0.020846687	GO:0051926	BP	negative regulation of calcium ion transport	Any process that stops, prevents, or reduces the frequency, rate or extent of the directed movement of calcium ions into, out of or within a cell, or between cells, by means of some agent such as a transporter or pore.
M11	7	0.020846687	GO:0098974	BP	postsynaptic actin cytoskeleton organization	A process that is carried out at the cellular level which results in the assembly, arrangement of constituent parts, or disassembly of cytoskeletal structures comprising actin filaments and their associated proteins in the postsynaptic actin cytoskeleton.
M11	8	0.047363653	GO:0038093	BP	Fc receptor signaling pathway	A series of molecular signals initiated by the binding of the Fc portion of an immunoglobulin to an Fc receptor on the surface of a signal-receiving cell, and ending with regulation of a downstream cellular process, e.g. transcription. The Fc portion of an immunoglobulin is its C-terminal constant region.
M11	9	0.048160823	GO:1903350	BP	response to dopamine	NA
M11	10	0.048160823	GO:1903351	BP	cellular response to dopamine	NA

© 2020. Muraoka S et al. Published online at http://www.aginganddisease.org/EN/10.14336/AD.2020.1007

SUPPLEMENTARY DATA

Supplementary Table 5. An area under the ROC curve (AUC) for 18 proteins included in the M10 module.

Gene name	single	t-tau	p-tau181
t-tau	0.638		
p-tau181	0.813		
VPS29	0.675	0.5	0.813
USP7	0.65	0.5	0.813
RNF112	0.613	0.5	0.813
PRRT2	0.837	0.837	0.837
NAPA	0.625	0.5	0.813
TUBB4A	0.675	0.5	0.813
RAB10	0.55	0.5	0.813
ATP6V1H	0.638	0.5	0.813
UCHL1	0.625	0.5	0.813
PLXNA4	0.763	0.5	0.938
MIF	0.7	0.5	0.813
CKB	0.738	0.5	0.813
RAB4A	0.65	0.5	0.813
CS	0.662	0.5	0.813
SNAP25	0.8	0.8	0.963
EFR3A	0.563	0.5	0.813
VAMP2	0.738	0.5	0.813
PCBD1	0.563	0.500	0.813

Supplementary Table 6. An area under the ROC curve (AUC) for 32 proteins included in the M11 module.

Gene name	single	t-tau	p-tau181
t-tau	0.638		
p-tau181	0.813		
PFKP	0.863	0.863	0.863
CSNK2A2	0.837	0.837	0.925
MAPK1	0.825	0.825	0.813
USP5	0.8	0.8	0.925
GNAO1	0.8	0.8	0.813
UBA1	0.775	0.775	0.938
NCEH1	0.775	0.775	0.813
SH3GL2	0.763	0.763	0.813
ITGA6	0.763	0.5	0.813
HSPH1	0.713	0.5	0.813

© 2020. Muraoka S et al. Published online at http://www.aginganddisease.org/EN/10.14336/AD.2020.1007

ACTG1	0.688	0.5	0.813
MON2	0.675	0.5	0.813
PRPS1	0.663	0.5	0.813
PON2	0.65	0.5	0.813
ATP6V1E1	0.638	0.5	0.813
CLTC	0.625	0.5	0.813
NDUFS1	0.625	0.5	0.813
HAGH	0.613	0.5	0.813
TUBA4A	0.6	0.5	0.813
CADPS	0.6	0.5	0.813
CNTN1	0.6	0.5	0.813
TUBA1A	0.588	0.5	0.813
PSMD2	0.575	0.5	0.813
VAT1L	0.563	0.5	0.813
NCAM1	0.562	0.5	0.813
HBB	0.55	0.5	0.813
PPIA	0.538	0.5	0.813
ATP1A2	0.538	0.5	0.813
DBN1	0.538	0.5	0.813
NCKIPSD	0.537	0.5	0.813
VAMP1	0.525	0.5	0.813
CNTNAP1	0.513	0.5	0.813

© 2020. Muraoka S et al. Published online at http://www.aginganddisease.org/EN/10.14336/AD.2020.1007
